# HTATIP2 regulates arteriogenic activity in monocytes from patients with limb ischemia

**DOI:** 10.1172/jci.insight.131419

**Published:** 2023-12-22

**Authors:** Ashish S. Patel, Francesca E. Ludwinski, Angeles Mondragon, Katherine Nuthall, Prakash Saha, Oliver Lyons, Mario Leonardo Squadrito, Richard Siow, Michele De Palma, Alberto Smith, Bijan Modarai

**Affiliations:** 1 Academic Department of Vascular Surgery, South Bank Section, School of Cardiovascular and Metabolic Medicine & Sciences, King’s BHF Centre of Research Excellence, King’s College London, United Kingdom.; 2 Swiss Institute for Experimental Cancer Research (ISREC), School of Life Sciences, École Polytechnique Fédérale de Lausanne (EPFL), Lausanne, Switzerland.; 3 Department of Vascular Biology and Inflammation, South Bank Section, School of Cardiovascular and Metabolic Medicine & Sciences, King’s BHF Centre of Research Excellence, King’s College London, United Kingdom.

**Keywords:** Angiogenesis, Therapeutics, Cardiovascular disease, Macrophages, Monocytes

## Abstract

Use of autologous cells isolated from elderly patients with multiple comorbidities may account for the modest efficacy of cell therapy in patients with chronic limb threatening ischemia (CLTI). We aimed to determine whether proarteriogenic monocyte/macrophages (Mo/MΦs) from patients with CLTI were functionally impaired and to demonstrate the mechanisms related to any impairment. Proarteriogenic Mo/MΦs isolated from patients with CLTI were found to have an impaired capacity to promote neovascularization in vitro and in vivo compared with those isolated from healthy controls. This was associated with increased expression of human HIV-1 TAT interactive protein-2 (*HTATIP2*), a transcription factor known to suppress angiogenesis/arteriogenesis. Silencing *HTATIP2* restored the functional capacity of CLTI Mo/MΦs, which was associated with increased expression of arteriogenic regulators Neuropilin-1 and Angiopoietin-1, and their ability to enhance angiogenic (endothelial tubule formation) and arteriogenic (smooth muscle proliferation) processes in vitro. In support of the translational relevance of our findings, silencing *HTATIP2* in proarteriogenic Mo/MΦs isolated from patients with CLTI rescued their capacity to enhance limb perfusion in the ischemic hindlimb by effecting greater angiogenesis and arteriogenesis. Ex vivo modulation of *HTATIP2* may offer a strategy for rescuing the functional impairment of pro–angio/arteriogenic Mo/MΦs prior to autologous delivery and increase the likelihood of clinical efficacy.

## Graphical abstract

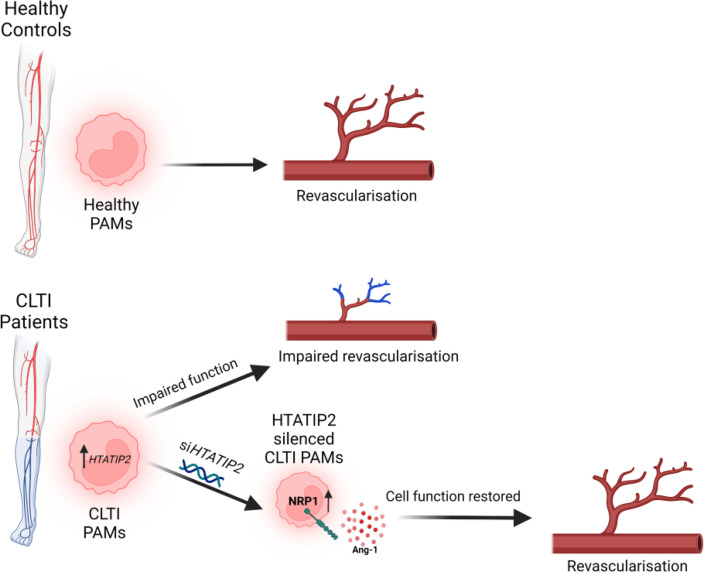

## Introduction

Peripheral arterial disease affects approximately 20% of individuals over the age of 75 and can lead to a severe restriction of blood flow to the lower limb, causing chronic limb threatening ischemia (CLTI) (1, 2). Patients with CLTI develop pain at rest and ulceration or gangrene, and up to a third will eventually require limb amputation (3, 4). This dire prognosis has led to increasing interest in alternative treatments for limb salvage, including cell therapies aimed at stimulating the growth of new blood vessels (5). Despite promising results in the laboratory, clinical trials have reported modest efficacy (6–9). Failed attempts to date have used mixtures of cells, a very small proportion of which had the potential to stimulate neovascularization (10–13). The fleeting vessels that appear after angiogenesis may be insufficient for supporting blood flow — rather, the robust circulation that is established by arteriogenesis may be essential for restoring limb perfusion (14). Functional impairment in autologous cells isolated from aged patients with multiple comorbidities may also account for the modest clinical results.

Proarteriogenic monocytes/macrophages (Mo/MΦs) expressing TIE2 promote neovascularization in the ischemic limb and are upregulated in patients with CLTI (15, 16). Phosphorylation of the TIE2 receptor by its angiopoietin agonists induces an activation of the downstream phosphokinases ERK and AKT not seen in TIE2^–^ Mo, while loss of signaling via this receptor attenuates the capacity of proarteriogenic Mo/MΦs in the ischemic hindlimb (15). A similar role has also been demonstrated for these cells in tumor neovascularization and growth (17–19). The relative ease by which proarteriogenic Mo/MΦs may be isolated from the circulation of patients makes them an attractive cell type for novel therapies. However, their upregulation in the circulation and ischemic limbs of patients with CLTI suggests that they are unable to effect spontaneous/sustained limb revascularization in this patient group (15). These autologous cells may be deleteriously affected by factors such as patient age, comorbidities, and the systemic effects of ischemia, thus limiting their endogenous function as well as their clinical utility for autologous therapeutic revascularization (20).

Ex vivo modulation of cells prior to their use has been proposed for enhancing the clinical efficacy of cell-based therapies. For example, mesenchymal stem cells can be preconditioned using either hypoxia or chemical agents prior to cell transplantation to improve their therapeutic efficacy in the target tissue, a strategy that is currently under investigation in clinical trials (21). This approach may be a strategy for enhancing the therapeutic potency of poorly functioning autologous cells ex vivo prior to delivery, circumventing the need for immunogenic allogeneic cells. This approach merits investigation in the context of proarteriogenic Mo/MΦs for the treatment of CLTI.

The aim of this study was to (a) compare the angiogenic and arteriogenic activity of proarteriogenic Mo/MΦs isolated from patients with CLTI with those from matched controls and investigate putative mechanisms for any differences seen and to (b) determine whether it is possible to rescue functional impairment in proarteriogenic Mo/MΦs through ex vivo modulation that initially involved in vitro mechanistic, and in vivo functional analyses, using Tie2-expressing immortalized murine BM–derived MΦs (iBMMs) (22, 23) under standard culture conditions. The findings from the iBMM experiments were subsequently tested using Mo/MΦs isolated from patients with CLTI.

## Results

### Proarteriogenic Mo/MΦs from patients with CLTI are functionally impaired

#### In vitro.

**Figure 1:**
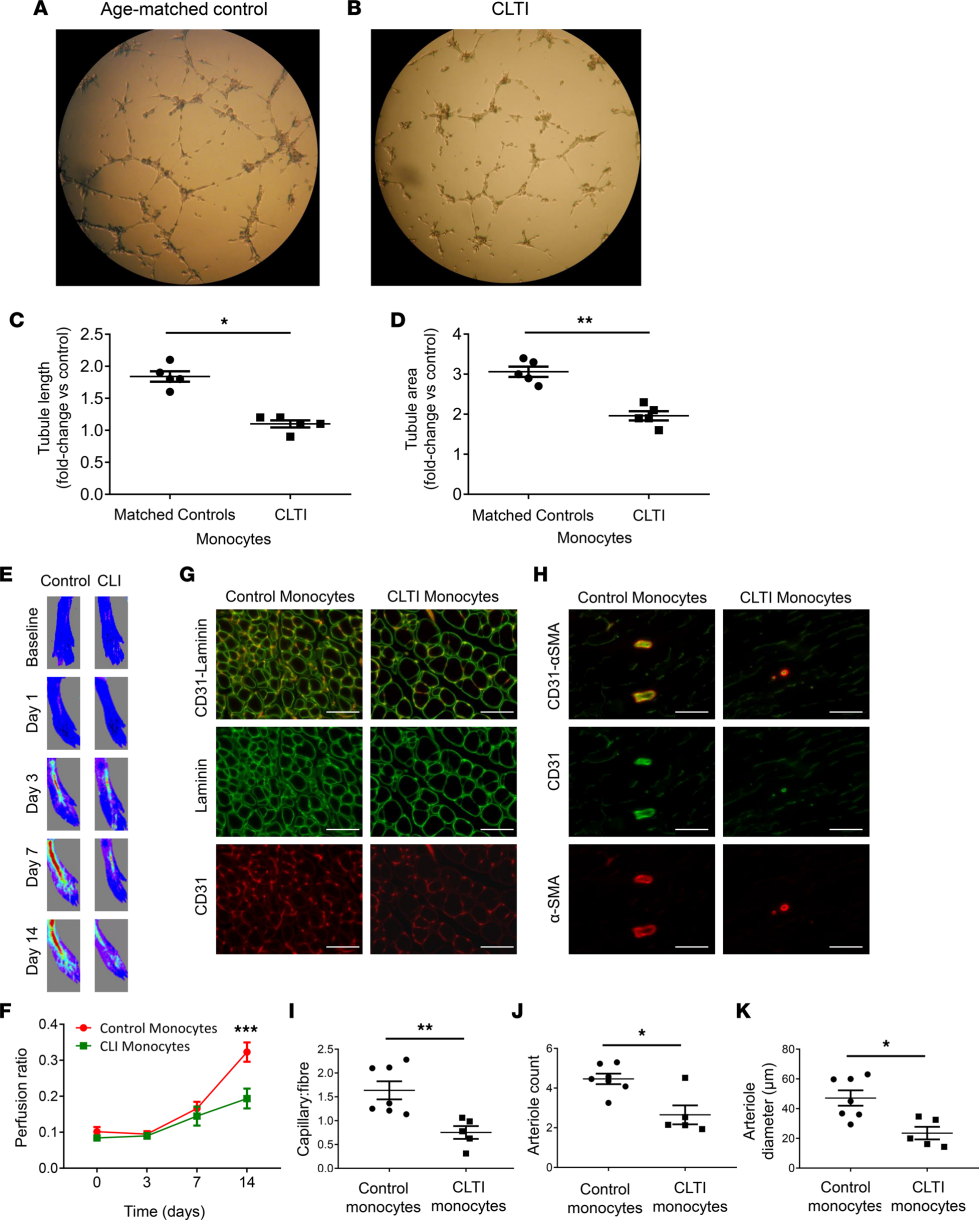
Reduced angiogenic and arteriogenic activity in proarteriogenic Mo/MΦs from patients with CLTI compared with age-matched controls. (**A** and **B**) Representative bright-field images of tubule formation following coculture of HUVECs with proarteriogenic Mo/MΦs isolated from age-matched controls (**A**) and patients with CLTI (**B**, *n* = 5/group). (**C** and **D**) The length (**C**) and area (**D**) of EC tubules formed in the coculture assay were quantified using an ImageJ macro. Fold-change in tubule expression is relative to that of assays containing HUVECs only. (**E**) Laser Doppler images of paw perfusion at days 3, 7, and 14 following induction of hindlimb ischemia in nude, athymic mice. (**F**) The ischemic limbs of mice were injected with proarteriogenic Mo/MΦs from controls (left) or patients with CLTI (right) (*n* = 7/group). Perfusion ratio calculated by comparison with contralateral limb. (**G**–**K**) Gastrocnemius muscle from the ischemic leg was analyzed for expression of CD31 (red) and laminin (green, **G**) and adductor muscle for α-SMA (red, **H**) to quantify capillary/fiber ratio (**I**) and α-SMA^+^ arteriole number (**J**) and diameter (**K**) (*n* = 5–7/group). *P < 0.05. Scale bar: 10μm. (**C**, **D**, **H**, **J**, and **K**) Data are presented as mean ± SEM. *P < 0.05, **P < 0.01 (Mann-Whitney *U* test). (**F**) Data were analyzed by 2-way ANOVA and post hoc Bonferroni test. ***P < 0.0001. Mo/MΦ, monocyte/macrophage; CLTI, chronic limb threatening ischemia; EC, endothelial cell; α-SMA, α-smooth muscle actin.

Comparison of the in vitro angiogenic activity of proarteriogenic Mo/MΦs isolated from patients with CLTI and age-matched controls using the HUVEC/Matrigel assay revealed reduced tubule length (1.10 ± 0.06 versus 1.84 ± 0.08 [*P* < 0.01]) and tubule area (1.96 ± 0.12 versus 3.06 ± 0.13 [*P* < 0.01]) when HUVECs were cocultured with CLTI proarteriogenic Mo/MΦs as opposed to control proarteriogenic Mo/MΦs (Figure 1, A–D).

#### In vivo.

Revascularization of ischemic murine hindlimbs was impaired in limbs injected with proarteriogenic Mo/MΦs from patients with CLTI compared with those injected with cells isolated from matched controls (Figure 1E). After 14 days, the perfusion index of limbs injected with proarteriogenic Mo/MΦs from patients with CLTI was 1.7-fold lower than those injected with control cells (*P* < 0.0001; Figure 1F). Histological analysis of capillary/fiber ratio (Figure 1G) and arteriole number and size (Figure 1H) in muscle from the ischemic limbs of these mice revealed reduced capillary/fiber ratio (0.8 ± 0.1 versus 1.6 ± 0.2, *P* < 0.01; Figure 1I), arteriole number (2.7 ± 0.5 versus 4.5 ± 0.3, *P* < 0.05; Figure 1J), and arteriole diameter (23.5 ± 4.2 μm versus 48.2 ± 5.2 μm, *P* < 0.05; Figure 1K) in mice treated with CLTI proarteriogenic Mo/MΦs.

#### HTATIP2 expression is upregulated in CLTI proarteriogenic Mo/MΦs.

**Figure 2:**
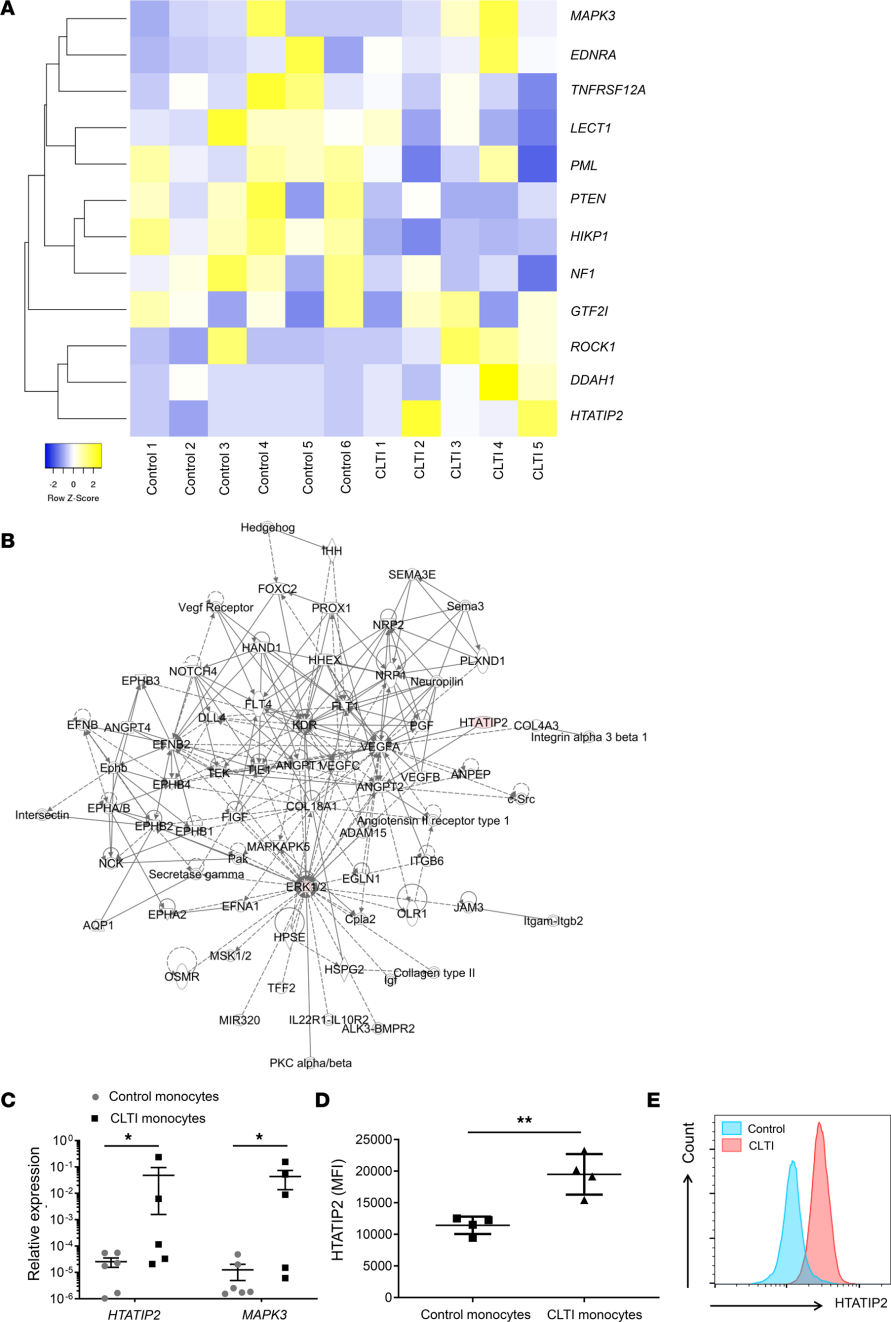
Differential mRNA and protein expression in proarteriogenic Mo/MΦs from patients with CLTI and matched controls. (**A**) Heatmap depicting the top differentially expressed genes associated with angio/arteriogenesis between matched control (*n* = 6) and CLTI patient (*n* = 5) proarteriogenic Mo/MΦs. Columns represent biological replicates, and rows represent genes. Gene expression is illustrated using pseudocolor scale (–2 to 2), with yellow representing high expression and blue representing low expression. Clustering between genes is determined using Euclidean distance metric. (**B**) Ingenuity Pathway Analysis of microarray data indicating significantly upregulated genes in CLTI proarteriogenic Mo/MΦs in red. (**C**) HTATIP2 and MAPK3 mRNA expression in proarteriogenic Mo/MΦs from patients with CLTI and age-matched controls was normalized to expression of RPLP0 and ACTB reference genes and log transformed (*y* axis). (**D** and **E**) HTATIP2 protein expression in CLTI and age-matched control proarteriogenic Mo/MΦs was quantified using flow cytometry and expressed in terms of median fluorescence intensity. Differential HTATIP2 expression is illustrated using histograms for age-matched control proarteriogenic Mo/MΦs (blue peak) and CLTI proarteriogenic Mo/MΦs (red peak, *n* = 4/group). (**C** and **D**) Data are presented as mean ± SEM. *P < 0.05 (Mann-Whitney *U* test), **P < 0.01 (unpaired *t* test). Mo/MΦ, monocyte/macrophage; CLTI, chronic limb threatening ischemia; HTATIP2, HIV-1 Tat interactive protein-2; ACTB, β-actin.

In order to elucidate a putative mechanism responsible for the reduced ability of CLTI proarteriogenic Mo/MΦs to promote the revascularization of ischemic muscle, we first sought to compare the whole-genome expression profiles of proarteriogenic Mo/MΦs isolated from patients with CLTI with those from matched controls. A total of 1,098 genes were differentially expressed, with fold difference > 3 (*P* < 0.05). The top differentially expressed genes are detailed in Supplemental Figure 1 and Supplemental Table 1 (supplemental material available online with this article; https://doi.org/10.1172/jci.insight.131419DS1). Since we wanted to identify aberrant expression of genes relating to angio/arteriogenic processes, we carried out functional gene analysis, specifically in the context of angio/arteriogenic regulation (Figure 2A and Supplemental Table 2). Subsequent Ingenuity Pathway Analysis (IPA) identified significant differential expression of *HTATIP2* and *MAPK3* between CLTI and matched control proarteriogenic Mo/MΦs (Figure 2B). These differences were confirmed by quantitative PCR (qPCR), with greater *HTATIP2* and *MAPK3* mRNA expression in CLTI proarteriogenic Mo/MΦs (*P* < 0.05; Figure 2C). HTATIP2 expression is associated with inhibition of vessel development in tumors (24); therefore, we sought to determine whether the expression of this protein is increased in proarteriogenic Mo/MΦs from patients with CLTI. Flow cytometric analysis of HTATIP2 protein expression in these cells from patients with CLTI and controls revealed an almost 2-fold greater HTATIP2 median fluorescence intensity (MFI) in CLTI cells (19.5 × 10^3^ ± 1.6 × 10^3^ versus 11.4 × 10^3^ ± 700, *P* < 0.01; Figure 2, D and E). This finding confirmed our microarray and qPCR data.

#### Inhibition of HTATIP2 expression in proarteriogenic Mo/MΦs enhances their angiogenic and arteriogenic potential.

**Figure 3:**
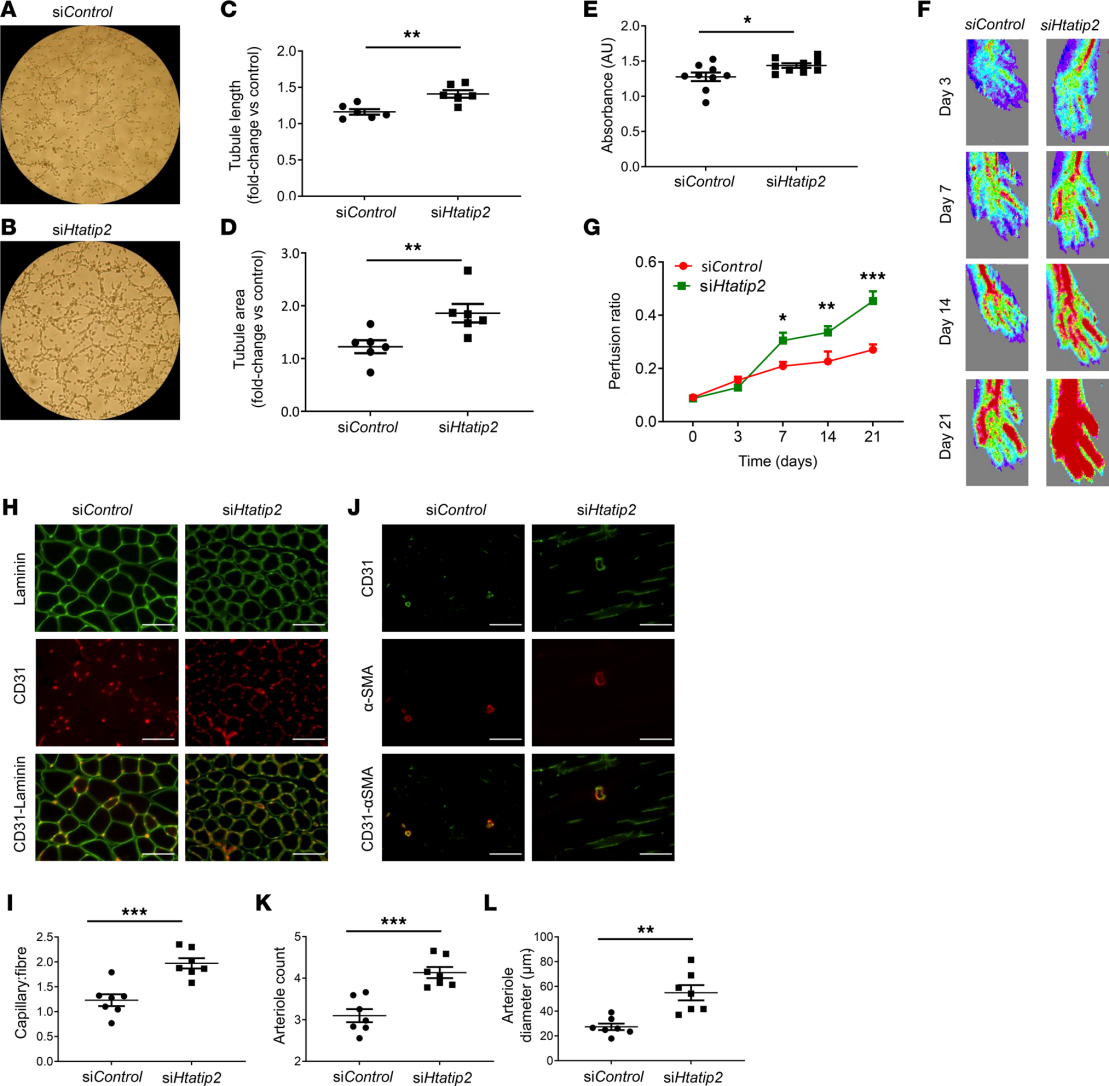
siRNA-mediated silencing of Htatip2 enhances the angio/arteriogenic function of proarteriogenic Mo/MΦs. (**A** and **B**) Representative bright-field images of endothelial cells cocultured with si*Control* siRNA or si*Htatip2* siRNA iBMMs (*n* = 6/group). (**C** and **D**) Quantification of EA.hy926 cell tubule length (**C**) and area (**D**) following coculture. Fold-change in tubule expression is relative to that of assays containing EA.hy926 cells only. (**E**) SMC proliferation was assessed when cells were exposed for 24 hours to conditioned media from si*Htatip2*- or si*Control*-iBMMs (*n* = 9/group). (**F** and **G**) The potential of Htatip2-silenced (*n* = 6) or si*Control*-iBMMs (*n* = 7) to promote reperfusion in the ischemic hindlimb of C57BL/6 mice was quantified by laser Doppler imaging over 21 days. *P* < 0.001 by repeated-measures 2-way ANOVA and *P < 0.05, **P < 0.01, ***P < 0.001 by post hoc Bonferroni test. (**H**–**L**) Histological analysis of ischemic limb muscle from si*Htatip2-* and si*Control-*iBMM–treated animals for CD31 (red) and laminin (green) staining (**H**) and α-SMA staining (**J**, red) to quantify capillary/fiber ratio (**I**), arteriole count (**K**), and arteriole diameter (**L**) respectively. Scale bar: 10 μm. (**C**–**E**, **I**, **K**, and **L**) Data are presented as mean ± SEM. (**C**–**E**) Data were analyzed by paired *t* test. *P < 0.05, **P < 0.01. (**H**–**L**) Data were analyzed by unpaired *t* test. **P < 0.01, ***P < 0.001. (**C** and **D**) Data are expressed as fold-change compared with tubule formation of EA.hy926 cells alone. (**G**) Data were analyzed by 2-way ANOVA and post hoc Bonferroni test. *P < 0.05, **P < 0.01, ***P < 0.0001. Mo/MΦ, monocyte/macrophage; iBMM, immortalized bone marrow macrophage; HTATIP2, HIV-1 Tat interactive protein-2; SMC, smooth muscle cell; α-SMA, α-smooth muscle actin.

We used siRNA silencing to examine whether suppression of *HTATIP2* expression would improve the neovascularization capacity of proarteriogenic Mo/MΦs. In order to standardize experimental conditions, we first used a cell line of iBMMs modified to overexpress the murine Tie2 receptor (22, 23). Comparisons were made between *Htatip2*-siRNA–treated iBMMs (si*Htatip2*-iBMMs) and control siRNA–treated iBMMs (si*Control*). A 77% reduction in *Htatip2* gene expression was achieved and maintained for at least 5 days in culture without affecting cell viability (measured by flow cytometric analysis of 7-AAD staining; Supplemental Figure 2). Analysis of the angiogenic activity of these cells in the HUVEC/Matrigel assay revealed enhanced tubule formation by si*Htatip2*-iBMMs compared with si*Control*-treated cells (Figure 3, A–D), with fold changes in tubule length and area of 1.41 ± 0.05 versus 1.16 ± 0.0.04 and 1.86 ± 0.18 versus 1.22 ± 0.12, respectively (*P* < 0.01 for both). We also investigated whether these cells secreted factors that would promote in vitro proliferation of SMCs as a measure of arteriogenic function. SMC proliferation measured by XTT assay was greater in cells exposed to si*Htatip2*-iBMM compared with si*Control* conditioned media (1.44 ± 0.03 versus 1.27 ± 0.06 AU, *P* < 0.05; Figure 3E). Delivery of si*Htatip2*-iBMMs into the hindlimb ischemia (HLI) model resulted in greater improvement in limb perfusion compared with si*Control*-treated limbs (*P* < 0.0001; Figure 3, F and G). Histological analysis of muscle from ischemic hindlimbs revealed ~60% greater capillary/fiber ratio (Figure 3H) in the gastrocnemius muscles of mice injected with si*Htatip2*-iBMMs compared with si*Control*-treated limbs (1.97 ± 0.10 versus 1.23 ± 0.12, respectively; *P* < 0.005; Figure 3I). Staining for arterioles (α-SMA^+^ vessels) in adductor muscle revealed significantly increased numbers of arterioles (4.1 ± 0.1 versus 3.1 ± 0.2/field of view, *P* < 0.0001; Figure 3, J and K) as well as a 2-fold increase in arteriole diameter (54.9 ± 6.1 μm versus 27.4 ± 2.6 μm, *P* < 0.005; Figure 3L) in mice treated with si*Htatip2*-iBMMs.

Since our in vitro and in vivo data highlight increased angiogenic and arteriogenic activity by *Htatip2*-silenced iBMMs, we sought to determine the mechanisms through which this occurs.

#### Increased expression of angiogenesis and arteriogenesis-associated proteins after Htatip2 silencing.

**Figure 4:**
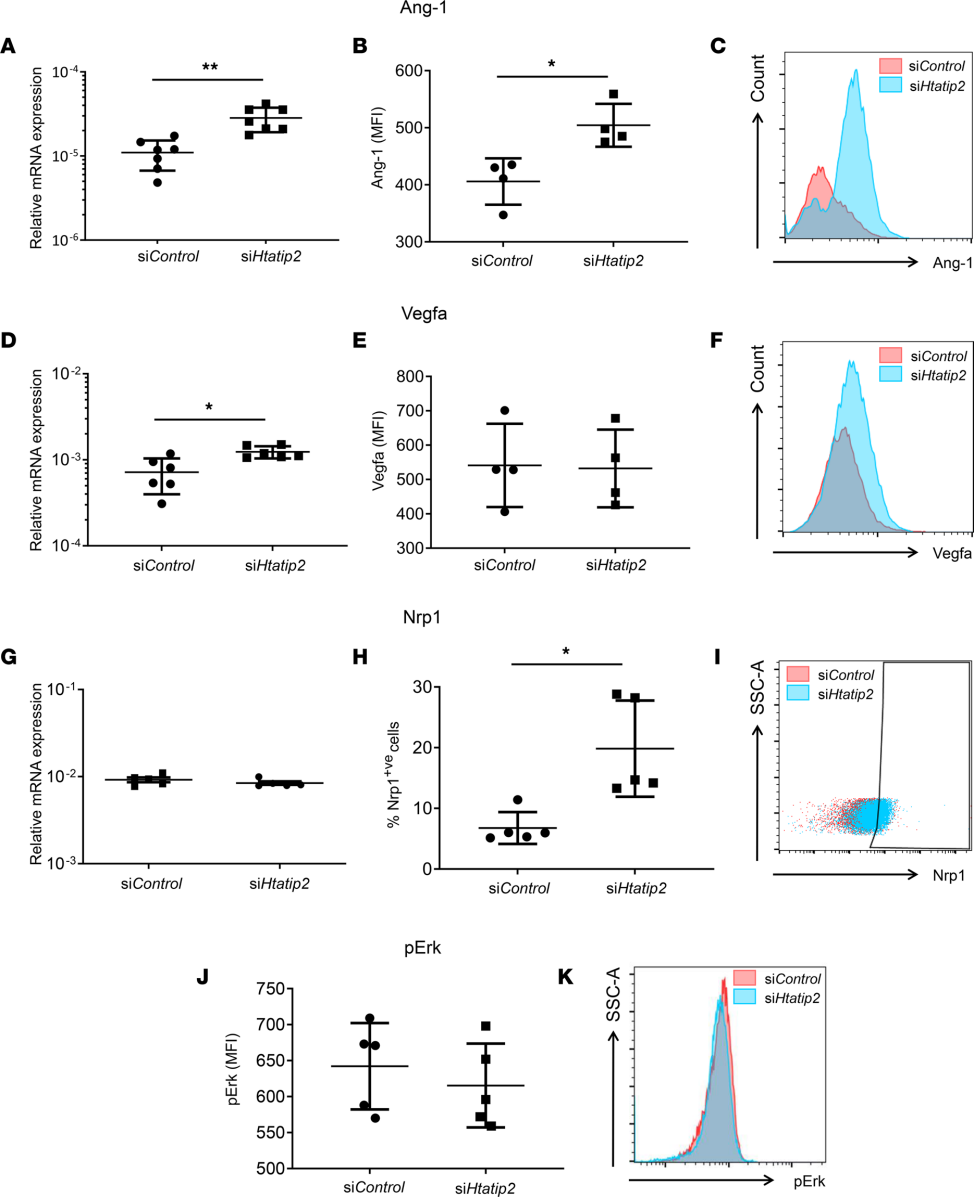
The effect of silencing Htatip2 on the activity of proarteriogenic Mo/MΦs. (**A**, **D**, and **G**) Angpt1, Vegfa, and Nrp1 mRNA expression quantification in si*Htatip2-* and si*Control*-iBMMs, normalized to Actb and Gapdh expression (*n* = 7/group). (**B**, **C**, **E**, **F**, **J**, and **K**) Intracellular levels of Ang-1, Vegfa and pERK protein in si*Htatip2-* and si*Control*-iBMMs measured by flow cytometry (*n* = 4/group). (**H** and **I**) Nrp1 protein expression quantified in si*Htatip2-* and si*Control*-iBMMs (*n* = 5/group) by flow cytometry (**I**) and expressed as the percentage of cells expressing Nrp1 (**H**). (**A**, **B**, **D**, **E**, **G**, and **H**) Data are presented as mean ± SEM. *P < 0.05, **P < 0.001 (paired *t* test). Mo/MΦ, monocyte/macrophage; iBMM, immortalized bone marrow macrophage; HTATIP2, HIV-1 Tat interactive protein-2; ACTB, β-actin; Ang-1, angiopoietin-1; Nrp1, neuropilin-1.

A number of factors modulate angio/arteriogenesis, including angiopoeitin-1 (Ang-1) and vascular endothelial growth factor-A (Vegfa) (25). Overexpression of Htatip2 in tumor cell lines has previously been shown to diminish expression of *Angpt1* mRNA (26). We assessed differences in the mRNA and protein expression of several angio/arteriogenesis-associated genes (Supplemental Figure 3). Silencing *Htatip2* in iBMMs resulted in an increase in both *Angpt1* mRNA (*P* < 0.01; Figure 4A) and Ang-1 protein expression (*P* < 0.05; Figure 4, B and C). VEGF-A/VEGF-receptor 2 (VEGFR2) and its coreceptor, neuropilin-1 (NRP1), are important for the generation of new blood vessels (27). Increased VEGFA mRNA (*Vegfa*) production has been reported in Htatip2-silenced tumor cells (26). We saw a differential expression of *Vegfa* mRNA following *Htatip2* silencing in iBMMs (*P* < 0.05; Figure 4D) but did not find a concomitant significant increase in Vegfa protein expression (Figure 4, E and F). Quantification of *Nrp1* mRNA expression revealed no significant difference in *Htatip2*-silenced cells (*P* > 0.05; Figure 4G); however, flow cytometric quantification of the Vegfa coreceptor Nrp1 on si*Htatip2*-iBMMs and si*Control*-treated cells (Figure 4, H and I) revealed a significant increase in Nrp1-expression following *Htatip2* silencing (19.8% ± 3.5% versus 6.8% ± 1.2% of cells, *P* < 0.01; Figure 4H). Silencing *Htatip2* did not affect downstream signaling via pErk (*P* > 0.05; Figure 4, J and K), which is known to be activated by VegfA to promote angiogenesis.

#### Enhanced angiogenic and arteriogenic function of siHtatip2-iBMMs is mediated by signaling via Nrp1 and Ang-1.

**Figure 5:**
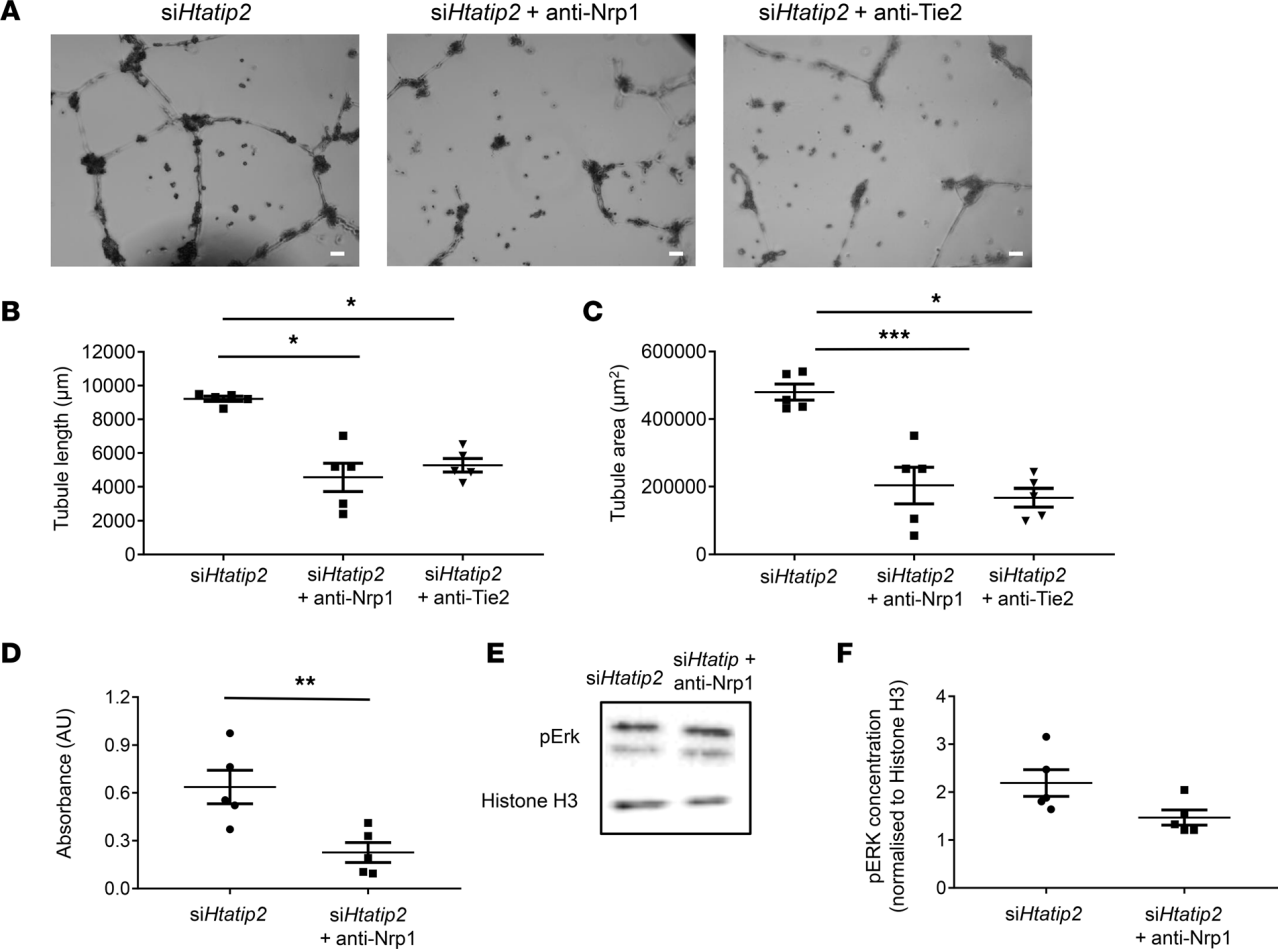
The functional significance of upregulated Ang-1 and Nrp1 expression in si*Htatip2* proarteriogenic Mo/MΦs. (**A**) Representative bright-field images of HUVECs cocultured with *Htatip2*-siRNA–transfected iBMMs with blockade of sAng-1 or iBMM Nrp1 receptor (*n* = 5/group). Scale bar: 100 μm. (**B** and **C**) HUVEC cell tubule length (**B**) and area (**C**) quantification following coculture with *Htatip2*-siRNA transfected iBMMs with blockade of sAng-1 or iBMM Nrp1 receptor (*n* = 5/group). (**D**) SMC proliferation following culture with conditioned media from si*Htatip2* iBMMs with and without iBMM Nrp1 blockade (*n* = 5/group). (**E** and **F**) Representative Western blot images of pErk and Histone H3 expression in protein lysate from si*Htatip2* iBMMs with and without blockade of iBMM Nrp1 receptor. pErk protein was quantified following normalization to Histone H3 reference protein densitometry (*n* = 5/group). (**B**–**D** and **F**) Data are presented as mean ± SEM. *P < 0.05, **P < 0.01, ***P < 0.001 (Holm-Sidak’s multiple-comparison test or unpaired *t* test). Mo/MΦ, monocyte/macrophage; iBMM, immortalized bone marrow macrophage; HTATIP2, HIV-1 Tat interactive protein-2; Ang-1, angiopoietin-1; Nrp1, neuropilin-1.

Blockade of Nrp1 receptor function in si*Htatip2*-iBMMs using an anti-Nrp1 antibody abrogated their enhanced angiogenic function in vitro. Coculture of Nrp1-blocked cells with endothelial cells (ECs) on Matrigel resulted in significantly reduced EC tubule length (4,566 ± 833 μm versus 9,218 ± 153 μm, *P* < 0.05; Figure 5, A and B) and area (2.0 × 10^5^ ± 0.5 × 10^5^ μm^2^ versus 4.8 × 10^5^ ± 0.2 × 10^5^ μm^2^, *P* < 0.05; Figure 5, A and C) compared with Nrp1-functioning si*Htatip2*-iBMMs. Conditioned media from cells with blocked Nrp1 receptor function induced less SMC proliferation than untreated si*Htatip2*-iBMMs (0.23 ± 0.09 AU versus 0.66 ± 0.10 AU, respectively; *P* < 0.05; Figure 5D). Blockade of Nrp1 receptor function in si*Htatip2*-iBMMs was also associated with reduced intracellular pERK signaling in response to VEGFA stimulation (Figure 5, E and F) compared with Nrp1-functioning si*Htatip2*-iBMMs (fold-change, 1.6 versus 2.1), but this did not reach statistical significance.

Blockade of soluble Ang-1 produced by si*Htatip2*-iBMMs decreased the formation of endothelial tubules (length: 5,277 ± 400 μm versus 9,218 ± 153 μm, *P* < 0.05; Figure 5, A and B; area: 1.7 × 10^5^ ± 0.3 × 10^5^ μm^2^ versus 4.8 × 10^5^ ± 0.2 × 10^5^ μm^2^, *P* < 0.05; Figure 5, A and C) compared with untreated si*Htatip2*-iBMMs.

#### Silencing HTATIP2 in proarteriogenic Mo/MΦs from patients with CLTI rescues their angiogenic and arteriogenic function in vitro.

**Figure 6:**
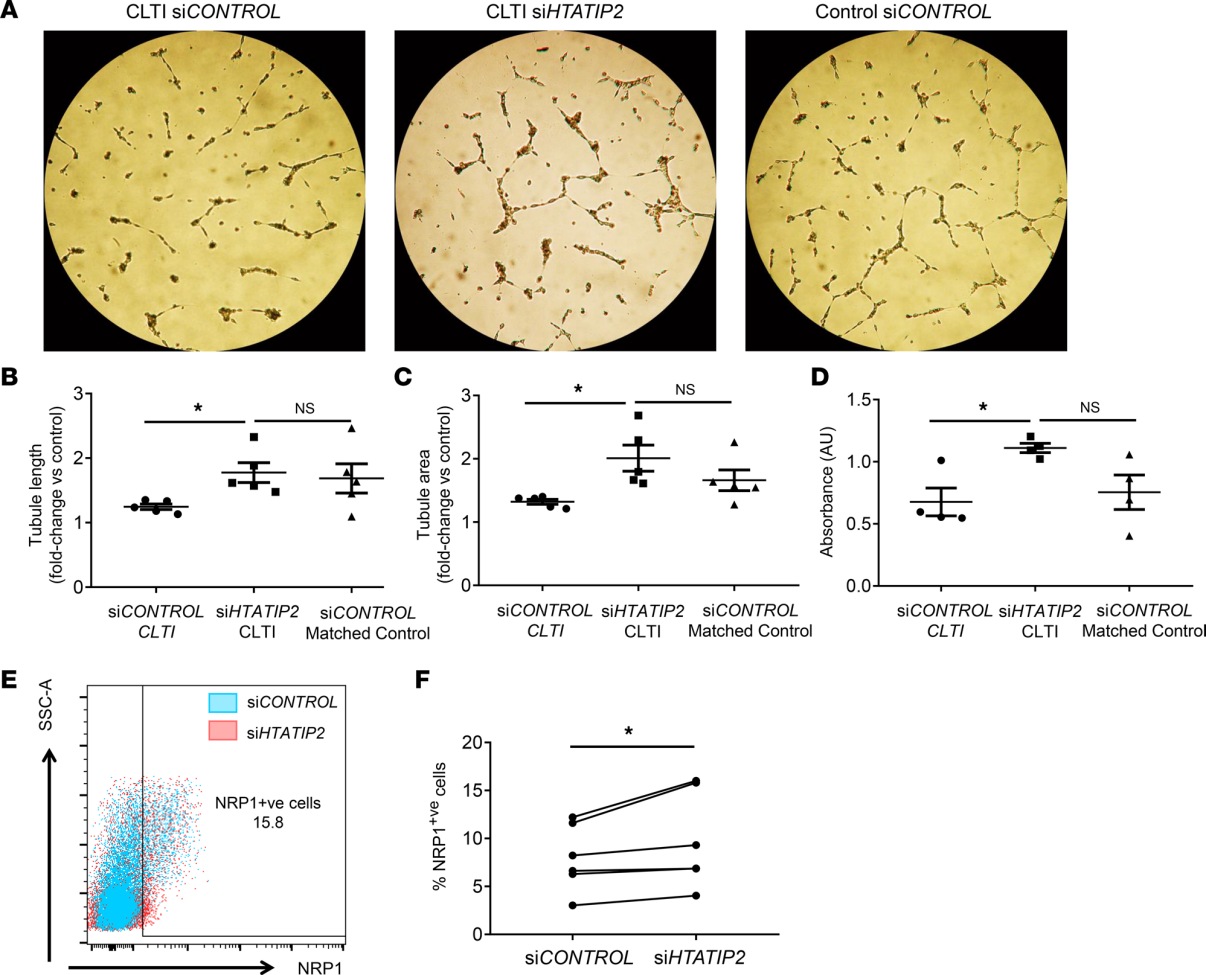
Silencing HTATIP2 expression in CLTI proarteriogenic Mo/MΦs enhances their angiogenic and arteriogenic function. (**A**) Representative bright-field images of HUVECs cocultured with si*CONTROL* siRNA or si*HTATIP2* siRNA silenced proarteriogenic Mo/MΦs from patients with CLTI and matched controls. (**B** and **C**) Quantification of HUVEC cell tubule length (**B**) and area (**C**) following coculture with si*HTATIP2*- or si*CONTROL*-silenced proarteriogenic Mo/MΦs (*n* = 5/group). (**D**) SMC proliferation following culture with conditioned media from si*HTATIP2*- or si*CONTROL*-silenced proarteriogenic Mo/MΦs isolated from patients with CLTI and matched controls (*n* = 4/group). (**E** and **F**) Cell-surface expression of NRP1 in si*HTATIP2-* and si*CONTROL*-silenced proarteriogenic Mo/MΦs measured by flow cytometry (**E**) and expressed as a percentage of NRP1^+^ cells (**F**, *n* = 6/group). (**B**–**D**) Data are presented as mean ± SEM. (**F**) Data are presented as paired values. *P < 0.05 (Wilcoxon rank test [**F**] or Tukey’s multiple-comparison test [**B**–**D**]). Mo/MΦ, monocyte/macrophage; CLTI, chronic limb threatening ischemia; HTATIP2, HIV-1 Tat interactive protein-2; NRP1, Neuropilin-1.

We investigated whether the impairment in angiogenic and arteriogenic function of CLTI Mo could be rescued by silencing *HTATIP2* expression (Figure 6A). We found greater tubule formation by si*HTATIP2* proarteriogenic Mo/MΦs compared with si*CONTROL* proarteriogenic Mo/MΦs from patients with CLTI (fold-change for length: 1.78 ± 0.15 versus 1.25 ± 0.04, *P* < 0.05, Figure 6B; area: 2.01 ± 0.21 versus 1.32 ± 0.04, *P* < 0.05, Figure 6C). Following silencing of *HTATIP2* on CLTI proarteriogenic Mo/MΦs, the tubule forming capacity of these cells was comparable to that induced by si*CONTROL* proarteriogenic Mo/MΦs from matched controls (length fold change 1.69 ± 0.23, *P* = 0.99; area fold-change 1.66 ± 0.16, *P* = 0.99).

Treatment of SMCs with conditioned media from si*HTATIP2* proarteriogenic Mo/MΦs from patients with CLTI resulted in greater SMC proliferation compared with control-siRNA treated cells (1.1 ± 0.04 AU versus 0.7 ± 0.11 AU, *P* < 0.05; Figure 6D), and this was again comparable with levels of SMC proliferation induced by proarteriogenic Mo/MΦs from matched controls. Analysis of NRP1 cell surface expression in si*HTATIP2* and si*CONTROL* proarteriogenic Mo/MΦs from patients with CLTI also revealed a significant increase in the percentage of NRP1-expressing cells following *HTATIP2* silencing (9.81 ± 5.0 versus 7.99 ± 3.5, *P* = 0.03; Figure 6, E and F). 

**Figure 7:**
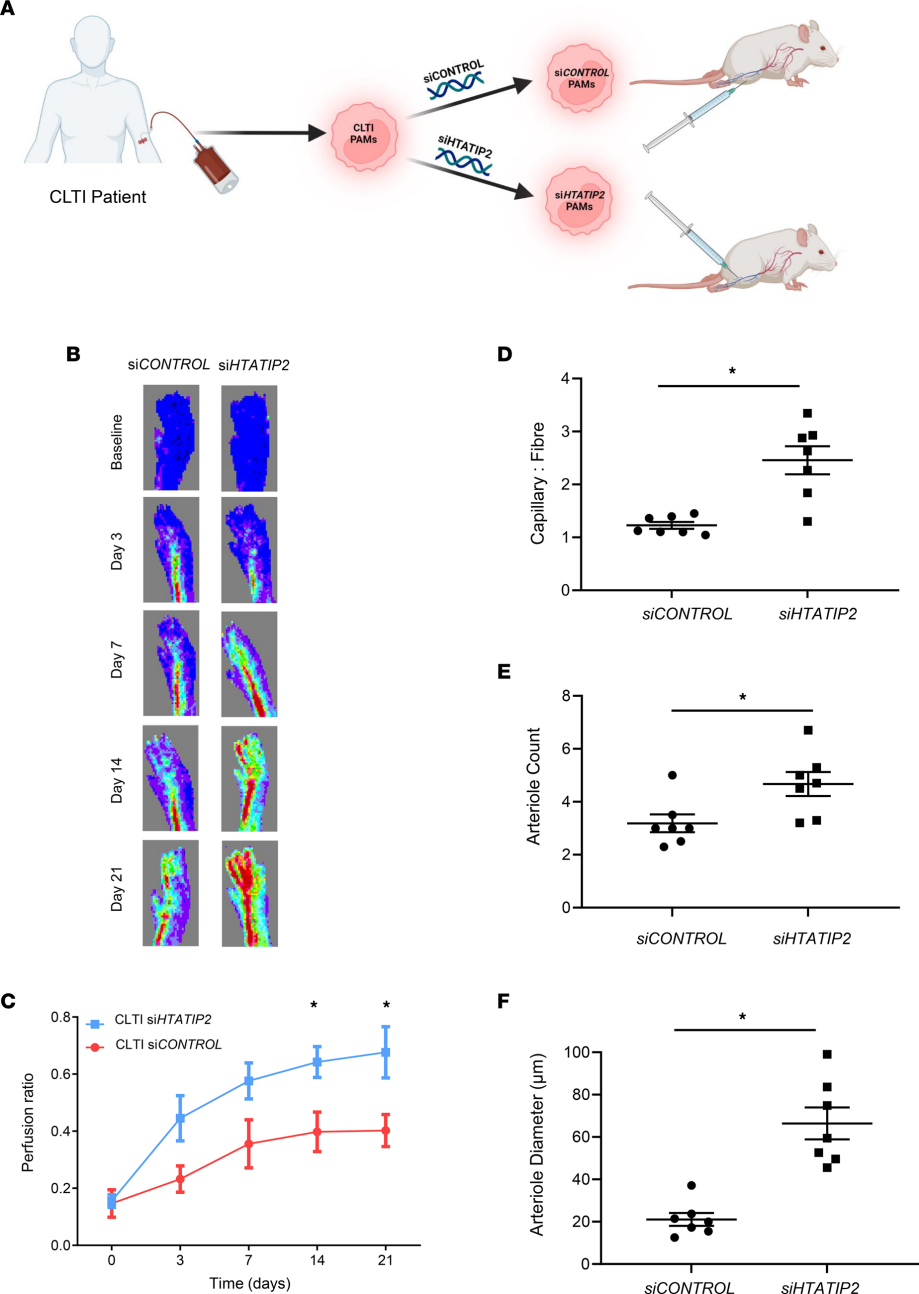
Silencing of *HTATIP2* on proarteriogenic Mo/MΦs from patients with *CLTI* rescues their angio- and arteriogenic function. (**A**) Proarteriogenic monocytes/macrophages (PAMs) were isolated from patients with CLTI, following by silencing with scrambled siRNA (si*CONTROL*) or *HTATIP2*-siRNA (si*HTATIP2*) and delivered into the ischemic limbs of nude, athymic mice. (**B** and **C**) The potential of *HTATIP2*-silenced and si*CONTROL* PAMs from patients with CLTI (*n* = 7/group) to promote revascularisation in nude, athymic mice was quantified by laser Doppler imaging over 21 days. *P < 0.05 by 2-way ANOVA and *P < 0.05 by post-hoc Bonferroni test. (**D**–**F**) Histological analysis of ischemic limb muscle from si*HTATIP2* and si*CONTROL* PAM-treated animals to capillary/fiber ratio (**D**), arteriole count (**E**), and arteriole diameter (**F**). Data are presented as mean ± SEM. (**D**–**F**) Data were analyzed by paired *t* test. *P < 0.05. Mo/MΦ, monocyte/macrophage; CLTI, chronic limb threatening ischemia; PAM, proarteriogenic monocyte/macrophage; *HTATIP2*, HIV-1 Tat interactive protein-2.

As a proof-of-concept experiment to support the translational potential of modulating *HTATIP2* in Mo/MΦs isolated from patients with CLTI, si*HTATIP2* proarteriogenic Mo/MΦs were compared with si*CONTROL* proarteriogenic Mo/MΦs in the HLI model in nude mice (Figure 7A). Injection of si*HTATIP2* proarteriogenic Mo/MΦs into the ischemic hindlimb resulted in an improvement in limb perfusion compared with si*CONTROL* proarteriogenic Mo/MΦs-treated limbs (*P* < 0.05; Figure 7, B and C). Histological analysis of muscle from ischemic hindlimbs revealed 2-fold greater capillary/fiber ratio in the muscles of mice injected with si*HTATIP2* proarteriogenic Mo/MΦs compared with si*CONTROL* proarteriogenic CLTI Mo/MΦs–treated limbs (2.46 ± 0.70 versus 1.23 ± 0.06, respectively; *P* < 0.05; Figure 7D). Staining for arterioles (α-SMA^+^ vessels) in adductor muscle revealed significantly increased numbers of arterioles (4.67 ± 0.46 versus 3.19 ± 0.34/field of view, *P* < 0.05; Figure 7E) as well as a 3-fold increase in arteriole diameter (66.41 ± 7.54 μm versus 21.07 ± 3.03 μm, *P* < 0.05; Figure 7F) in mice treated with si*HTATIP2* proarteriogenic CLTI Mo/MΦs.

## Discussion

The present study demonstrates that proarteriogenic Mo/MΦs isolated from patients with CLTI have approximately half the stimulatory capacity to induce angiogenesis and arteriogenesis when compared with the same cells isolated from matched controls. In order to determine a possible cause for impairment in these cells, we carried out a comparative whole-genome microarray analysis of proarteriogenic Mo/MΦs isolated from patients with CLTI, compared with those isolated from matched controls. Although angiogenesis-regulating genes were not among those most differentially expressed, a focused IPA on genes regulating angio/arteriogenesis followed by validation using qPCR identified raised expression in proarteriogenic Mo/MΦs from patients with CLTI of a gene for the transcriptional cofactor *HTATIP2* (also known as *TIP30* or *CC3*), which is known to inhibit neovascularization in tumors (24). The fact that our microarray identified other genes with larger differential expression between proarteriogenic Mo/MΦs from patients with CLTI and matched controls is not surprising, since these cells perform a variety of pathophysiological functions other than blood vessel development. Given the chronicity and range of comorbidities experienced by patients with CLTI, it would therefore not be unreasonable to expect a general effect on the expression of genes regulating many of the functions of Mo/MΦs. The evidence gathered from literature highlighting the potential antiangiogenic role of *HTATIP2* expression in tumor biology fits mechanistically with our data. We therefore focused on elucidating the effect of the upregulated expression of this tumor suppressor found in functionally impaired CLTI proarteriogenic Mo/MΦs. Although our pathway analysis revealed significantly increased *MAPK3* (*ERK1*) expression in CLTI proarteriogenic Mo/MΦs, in addition to upregulated *HTATIP2*, there is speculation as to the relevance of differential expression of a single ERK isoform, with the suggestion that changes in global *ERK* quantity are more pertinent with regard to affecting cell phenotype (28). We also did not investigate whether differential expression of *ERK1* affected the phenotype of CLTI proarteriogenic Mo/MΦs, since we did not demonstrate significant differences in the mRNA expression of other *ERK* isoforms.

Reduced *Htatip2* expression increases tumor vascularization in mice (24), while forcing the expression of HTATIP2 in tumor cell lines inhibits their ability to promote EC proliferation and migration (26). We therefore investigated whether inhibiting *HTATIP2* expression in proarteriogenic Mo/MΦs would enhance/rescue their ability to stimulate angio/arteriogenesis in vitro and in vivo. We used a well-characterized murine Tie2–expressing MΦ cell line (22) to investigate the effect of siRNA silencing of *Htatip2* on their activity. This resulted in an enhanced angiogenic and arteriogenic phenotype in vitro and translated to significantly improved reperfusion following injection of *Htatip2*-silenced proarteriogenic Mo/MΦs into the ischemic murine hindlimb. This was not only associated with increased angiogenesis but also, more notably, with arteriogenesis — a crucial mechanism for sustained improvements in perfusion (29, 30). We suggest that, despite upregulation of their numbers in the circulation in response to ischemia, the raised expression of *HTATIP2* in CLTI proarteriogenic Mo/MΦs may, in part, account for the reduced potency of these cells in stimulating angio/arteriogenesis in these patients. Although *Htatip2* silencing was sustained for only 5 days, delivery of these cells immediately following induction of ischemia provides sufficient time for the cells to exert their potent revascularizing effects (as early as 48 hours), as previously reported (15).

In search of a mechanism for how HTATIP2 may influence the angio/arteriogenic activity of Mo/MΦs, we silenced the expression of *Htatip2* using siRNA and found that this resulted in upregulated expression of the angio/arteriogenesis-associated VEGFR2 coreceptor Nrp1 and an ensuing increase in the angio/arteriogenic capacity of the cells. Blockade of signal transduction via Nrp1 on these cells abrogated this potent neovascularizing phenotype. Although Nrp1 expression on ECs is dispensable for VEGF/VEGFR2 signaling in the context of angiogenesis, it is crucial for arteriogenesis, where VEGF-driven phosphorylation of ERK determines arterial fate specification (31). In the absence of NRP1, and in the presence of high levels of VEGF/VEGFR2, activated VEGFR2 is internalized and trafficked to Rab7^+^ endosomes, resulting in rapid lysosomal degradation of the receptor (32). In the presence of NRP1, signal transduction is prolonged through Rab11^+^ endosome-mediated recycling of VEGFR2 to the cell surface (33). This ensures prolonged VEGF signaling and greater phosphorylation of ERK that confers a more arteriogenic stimulus (33, 34).

The role of NRP1/VEGFR2 signaling in MΦs is less well characterized. M2-like MΦs express higher levels of NRP1 and induce greater collateral vessel formation compared with M1-like MΦs (35, 36). NRP1 expression in tumor-associated MΦs is reduced in hypoxia, and angiogenesis is inhibited through prevention of MΦ migration (37). Our data show that blocking Nrp1 signal transduction in proarteriogenic Mo/MΦs suppresses Erk phosphorylation (which is normally induced by Vegfa-stimulation of Nrp1), thus abrogating the angiogenic and arteriogenic effects of *Htatip2* suppression in these cells.

Increased expression of HTATIP2 is associated with decreased expression of Ang-1 in tumor cells (26). Arteriole formation is diminished in mice with Ang-1–deficient SMCs, highlighting a potential role for Ang-1 in collateralization (38). In addition, stimulating MΦs with Ang-1 promotes α-SMA^+^ vessel formation following HLI (16). We show that si*Htatip2*-iBMMs have increased expression of Ang-1, resulting in enhanced SMC proliferation in a paracrine fashion.

It is important to recognize that other potential VEGF-A or TIE2 independent mechanisms may also promote angiogenesis upon silencing of HTATIP2. The role of other differentially expressed genes between matched controls and patients with CLTI, in particular Rho-associated coiled-coil kinase 1 (ROCK 1) and dimethylarginine dimethylaminohydralase-1 (DDAH-1), appear compelling and merit investigation in future studies (39, 40).

Aging and atherosclerosis, a hallmark of patients with CLTI, are known to affect the vascular regenerative niche where chronic inflammation and oxidative stress affects cell migration, mobilization, proliferation, signaling, and secretome production (41, 42). Previous studies have specifically shown impaired M2 MΦ function in aged rats (43).

Functional impairment is considered a key reason for the absence of clinical efficacy associated with autologous cell therapies, but hitherto, there has been a paucity of mechanistic data to support this assumption. Ex vivo preconditioning can mitigate functional impairment and potentiate the regenerative capacity in cell types, including bone marrow mononuclear cells and mesenchymal stem cells (44, 45). Similarly, the present study demonstrates that silencing HTATIP2 in pro–angio/arteriogenic Mo isolated from patients with patients with CLTI results in a striking increase in their capacity to revascularize the ischemic hindlimb, highlighting the translational potential of using this strategy for enhancing the therapeutic efficacy of these autologous cells.

In conclusion, the capacity of proarteriogenic Mo/MΦs from patients with CLTI to promote vessel formation is impaired by an increased expression of *HTATIP2* in these cells. We have demonstrated that silencing HTATIP2 expression in CLTI proarteriogenic Mo/MΦs rescues their arteriogenic potential, both in vitro and, crucially, in vivo. This identifies a strategy that could provide an opportunity for ex vivo preconditioning of these cells for clinical use.

## Methods

### Patients

Recruited patients with CLTI were those with rest pain and/or lower limb tissue loss (categorized according to Rutherford classification 4–6) (46). Control patients were matched for age and were included if they had no history or clinical evidence of peripheral vascular disease. CLTI and control patients with a history of diabetes, chronic renal failure (stages 4 and 5, GFR < 29 mL/min/1.73m^2^), malignancy, or steroid therapy were excluded. Each sample assayed was isolated from a single CLTI patient or matched control. Patient data pertaining to the microarray samples are given in Supplemental Table 5.

### Mice

C57BL/6 and BALB/c nude mice were purchased from Charles River Laboratories. All experimental animals were male and aged 8–10 weeks. Animals were housed in pathogen-free facilities at King’s College London. Animals were randomly allocated to experimental groups, and operators were blinded to this allocation.

### Cell lines and primary cultures

All in vitro cultures were performed under standard conditions (37°C, 21% O_2_, 5% CO_2_). HUVEC (Promocell) and EA.hy926 cells (ATCC) were cultured in endothelial basal media supplemented with 1 μg/mL hydrocortisone, 100 μg/mL penicillin, 100 μg/mL streptomycin sulphate, 250 ng/mL amphotericin B, 10 ng/mL recombinant human EGF, 3 ng/mL bFGF, 3 μg/mL heparin, and 2% FCS (Promocell). Tie2-iBMMs (22, 23). were cultured in complete medium (IMDM [Thermo Fisher Scientific], 20% FCS, 2 mM glutamine, 1% [v/v] antibiotic/antimycotic, 50 ng/mL MΦ CSF [M-CSF, Peprotech]). Tie2-iBMMs were transfected with siRNA/miRNA in serum-free iBMM media (IMDM, 1% antibiotic/antimycotic, 50 ng/mL murine M-CSF), and human cell transfection media consisted of serum-free RPMI1640 (MilliporeSigma), supplemented with 1% antibiotic/antimycotic and 1% L-glutamine. For generation of cell-conditioned media, transfected Tie2-iBMMs and primary human Mo were cultured in normal iBMM (IMDM, 20% heat-inactivated [HI] serum, 1% antibiotic/antimycotic, 50 ng/mL M-CSF) and normal Mo media (RPMI1640, 10% HI serum, 1% L-glutamine, 1% antibiotic/antimycotic), respectively. Human aortic smooth muscle cells (SMCs) were expanded in SmGM-2 SMC growth medium-2 (Lonza). Human dermal fibroblasts (Lonza) were cultured in DMEM (MilliporeSigma) supplemented with 10% FCS and 1% antibiotic/antimycotic.

### Proarteriogenic Mo/MΦ isolation

We used TIE2 as a marker of proarteriogenic Mo/MΦs, and cells were isolated according to previously published methodology (Supplemental Figure 4) (15). Briefly, peripheral blood mononuclear cells were isolated from 100 mL of venous blood by density centrifugation (at 400*g*, spun for 30 minutes at 4°C) using Ficoll-Paque (GE Healthcare). Whole-population Mo were magnetically isolated using anti-CD14 microbeads (Miltenyi Biotec) followed by FACS for proarteriogenic Mo/MΦs (Aria II; BD Biosciences). The following antibodies were used for cell sorting: human anti-TIE2 (R&D Systems), anti-CD14, anti-CD2, anti-CD3, anti-CD19 and anti-CD56 antibodies (BD Biosciences) (Supplemental Table 10).

### In vitro angiogenesis assay

The in vitro angiogenesis assay has been reported previously (15). HUVECs and EA.hy926 cells were expanded in culture using endothelial basal media supplemented with 1 μg/mL hydrocortisone, 100 μg/mL penicillin, 100 μg/mL streptomycin sulphate, 250 ng/mL amphotericin B, 10 ng/mL recombinant human EGF, 3 ng/mL bFGF, 3 μg/mL heparin, and 2% fetal calf serum (Promocell). Prior to the coculture assays, cells were serum starved in EC basal media (Promocell) for 6 hours. In total, 2 × 10^4^ cells were then seeded into each well of a μ-slide angiogenesis plate (Ibidi), which had been precoated with growth factor-reduced Matrigel (BD Biosciences), and cocultured with 5 × 10^4^ human Mo or siRNA-transfected iBMMs. Cells were cultured for 18–24 hours. Phase contrast microscopy was used to photograph endothelial tubule formation. Tubule length and area were quantified using automated image processing (Image-Pro Plus, Media Cybernetics) or using measurement functions in NIS Elements BR 4.20 software.

### Murine model of HLI

All murine surgeries were carried out under 3% gaseous isoflurane anesthesia in 1.5% O_2_ and in accordance with Home Office regulations. A severe model of ischemia was surgically induced in the hindlimb of either 8- to 10-week-old male nude, athymic (strain no. 194, Charles River Laboratories) or C57BL/6 mice (strain no. 000664, Charles River Laboratories) by ligation of the femoral artery proximal and distal to the profunda femoris branch and by excision of the intervening arterial segment. Human Mo (5 × 10^5^ cells/animal) or siRNA-transfected iBMMs (7.5 × 10^5^ cells/animal) were resuspended in 60 μL sterile PBS and delivered via single injection into the adductor muscle adjacent to the excised segment of artery immediately following HLI. This represents the maximum volume of cell suspension that is feasible to inject into the ischemic limb without inducing local tissue damage or injected cell viability. Improvements in perfusion have been reported as early as 48 hours following delivery of proarteriogenic Mo/MΦs in this way (15). Laser Doppler scanning (Moor Instruments) was performed immediately after surgery to confirm the induction of HLI. Mice were subsequently scanned at days 3, 7, and 14 (for nude, athymic mice) or 3, 7, 14, and 21 (for C57BL/6) mice. The perfusion ratios between the ischemic and contralateral paws were measured by blinded operators using mLDI Main v5.3 software (Moor Instruments). Adductor (proximal) and gastrocnemius (distal) muscle samples harvested from ischemic limbs were fixed in 4% paraformaldehyde for 30 minutes at 4°C and subsequently dehydrated using increasing concentrations of sucrose solution (15%, 30%, and 40% every 24 hours) for 72 hours. Specimens were embedded in OCT and snap frozen in ice-cold isopentane before sectioning.

### Histological analysis

Consecutive 10 μm cryosections were taken at 3 levels up to 250 μm apart. Sections were stained for laminin, CD31, and α-smooth muscle actin. Images were captured using a Nikon Ti Eclipse microscope and CoolSNAP HQ2 camera, and they were analyzed with NIS Elements BR 4.20 software. For CD31/laminin and α-SMA staining, 3 fields of view were captured per tissue section for analysis. At least 3 fields of view were analyzed from 3 sections at each level (providing 9 images per level and 3 levels for analysis). Capillary/fiber ratio was calculated by dividing the number of muscle fibers by the number of CD31^+^ capillaries per field of view. The diameters of α-SMA^+^ arterioles in each section were measured using measurement functions in the NIS Elements BR 4.20 software.

### Gene expression microarray

Circulating proarteriogenic Mo/MΦs were isolated from 5 patients with CLTI and 6 age-matched controls (Supplemental Table 5) by FACS as described previously (15), and mRNA extracted from 30,000 cells was obtained from each patient. The integrity of the generated cDNA was confirmed by picochip analysis (Agilent 2100 Bioanalyzer), and the mRNA was amplified according to in-house protocols and was reverse transcribed. cDNAs were hybridized to an Agilent Whole Human Genome Oligo Microarray 8x60K v2 following Cy3-labeling and fluorescence signals detected using Agilent’s Microarray Scanner System (Agilent Technologies).

### Microarray analysis

Quality control analysis of the array was carried out using Agilent Feature Extraction software to determine variation in mean signal across samples, the percentage of nonuniform outliers, and the number of found features in order to ensure comparability between samples. All samples passed the criteria for these parameters. Hierarchical clustering analysis and principal component analysis was performed using Partek Genomics Suite to assess integrity of the expression data. Reliably detected transcripts were identified based on transcript variance (threshold 0.01). Differential gene expression levels were determined by 2-way ANOVA analysis of normalized array values. The top 20 differentially expressed genes between CLTI patient and matched control proarteriogenic Mo/MΦs are listed in Supplemental Table 1. Functional enrichment analysis was performed to identify mechanisms underlying phenotypic differences between proarteriogenic Mo/MΦs from patients with CLTI and proarteriogenic Mo/MΦs from matched controls. The Ingenuity Knowledge Base and Gene Ontology database were searched for genes associated with angiogenesis. Those common between the two (547 genes) were selected for analysis. Those with differential expression between CLTI patient and matched control Mo/MΦs are detailed in Supplemental Tables 2–4. Functional gene networks were generated by overlaying the differential gene expression information with the top network of angiogenesis-associated genes.

### Proarteriogenic Mo/MΦ cell line generation

Previous research has demonstrated the potent pro–angio/arteriogenic potential of primary human and murine proarteriogenic Mo/MΦs (15). For proof of concept, we have induced TIE2 expression in a murine MΦ cell line that has been shown to possess angiogenic activity (Supplemental Figure 5). iBMMs were transduced with a lentiviral vector to express the murine Tie2 receptor (22, 23). Briefly, vesicular stomatitis virus–pseudotyped, third-generation lentiviruses were produced by plasmid transfection of 293T cells. The SV40 large T antigen coding sequence was cloned into the spleen focus-forming virus (SFFV) promoter–containing lentivirus using BamHI and SaII restriction enzymes, and the resultant lentivirus was used for transduction. Tie2 expression was subsequently induced via a second lentiviral transduction using a Tie2-expressing lentivirus. Tie2-iBMMs were cultured in complete medium (IMDM [Thermo Fisher Scientific], 20% FCS, 2 mM glutamine, 1% [v/v] antibiotic/antimycotic, 50 ng/mL M-CSF [Peprotech]).

### qPCR quantification of mRNA/miRNA expression

#### Microarray validation.

qPCR analysis was conducted on a 7900HT system (Applied Biosystems Inc) using SYBR Green assays (Eurogentec). Target primers were used at a final concentration of 200 nmol/L. A total of 4 ng/well of each cDNA sample was analyzed in triplicate for the genes of interest, and data were normalized to the expression of reference genes *RPLP0* and *ACTB* using the 2^–ΔΔCt^ method (47). Primer sequences are detailed in Supplemental Table 6.

#### Expression of angio/arteriogenic mediators following Htatip2 silencing.

For validation of siRNA-mediated silencing of *Htatip2* and assessment of the effect of this silencing on iBMM phenotype, predesigned single-tube assays against murine *Actb*, *Gapdh*, *Htatip2*, *Cd44*, *Fgf2*, *Icam1*, *Pdgf-b*, *Plgf*, and *Egf* with Universal TaqMan Mastermix were used (Invitrogen). A total of 5 ng/well of each cDNA sample was analyzed in triplicate, and expression of target genes was normalized to that of the reference genes *Actb* and *Gapdh*. The percentage of *Htatip2* silencing was determined by comparing *Htatip2* expression in cells with that of cells treated with control siRNA.

### HTATIP2 expression analysis by flow cytometry

HTATIP2 protein expression of isolated Mo/siRNA-treated Tie2-iBMMs was analyzed by flow cytometry. For human proarteriogenic Mo/MΦs, following FcR blocking (Miltenyi Biotec), cells were stained for surface expression of CD14 (BD Biosciences) and TIE2 (R&D Systems). Washed cells were subsequently fixed in 2% paraformaldehyde for 15 minutes at room temperature, permeabilized with Perm Buffer IV for 20 minutes (BD Biosciences), and stained for intracellular HTATIP2 (Abcam). Secondary antibody staining was then conducted using an APC-conjugated secondary antibody (Jackson ImmunoResearch) (Supplemental Table 10). Cells were subsequently washed, resuspended in buffer (PBS, 0.5% BSA, 2 mM EDTA), and analyzed using a MACSQuant flow cytometer (Miltenyi Biotec). Intracellular protein expression was quantified in terms of MFI using FlowJo V10 software.

### Inhibition of Htatip2 expression in iBMMs

iBMMs (2.5 × 10^5^) were seeded onto a 24-well plate and cultured for 18 hours in transfection media, which consisted of serum-free iBMM media (Iscove Modified Dulbecco Media [IMDM]with L-glutamine and 25 mM HEPES [Thermo Fisher Scientific, 12440-053], 1% antibiotic/antimycotic [Thermo Fisher Scientific, 15240-062], 50 ng/mL murine M-CSF [Peprotech, 315-02]), Hiperfect Transfection Reagent (Qiagen), and 100 nmol/L siRNA targeting *Htatip2* or a nonspecific control sequence (sequences detailed in Supplemental Table 7). RNA was extracted from cultured cells (Qiagen RNeasy mini kit, Qiagen) and reverse transcribed (High Capacity RNA-to-cDNA kit, Invitrogen). *Htatip2* knockdown was confirmed by qPCR using predesigned single-tube assays (Invitrogen). In total, 76.8% *Htatip2* mRNA silencing was achieved when compared with *Htatip2* expression in iBMMs transfected with si*Control* siRNA (Supplemental Figure 2). Cell viability following transfection was assessed using flow cytometry; < 5% cell death was observed for transfections with si*Htatip2* and si*Control* siRNA (Supplemental Figure 2). The duration of *Htatip2* silencing, determined by qPCR and flow cytometry, was 3–7 days (with 75% and 50% silencing, respectively; Supplemental Figure 2).

### Inhibition of HTATIP2 expression in human Mo

In total, 1.5 × 10^6^ Mo were isolated as outlined previously (15), seeded into a 6-well plate, and silenced for 48 hours according to published methodology using Hiperfect Transfection Reagent (6% v/v, Qiagen) and 100 nmol/L siRNA targeting *HTATIP2* or a nonspecific control sequence (sequences detailed in Supplemental Table 8) (48). *HTATIP2* silencing was confirmed by qPCR, and viability was assessed using flow cytometry as outlined previously. Totals of 77.2% *HTATIP2* mRNA silencing and < 3% cell death were achieved compared with si*CONTROL*-transfected Mo.

### 7-AAD staining for cell viability

Following siRNA transfection, cells were lifted off culture plates using trypsin before being washed and resuspended in 100 μL PBS containing 0.5% BSA 2 mmol/L EDTA. In total, 5 μL 7-AAD (BD Biosciences) was added to each sample, and samples were incubated for 15 minutes. Staining for 7-AAD was quantified using flow cytometry (MACSQuant, Miltenyi Biotec), using unstained cells to set the positive gate.

### Flow cytometry for Nrp-1, Ang-1, and Vegfa

Nrp1 protein expression was quantified using flow cytometry in si*Htatip2* siRNA–silenced and si*Control* siRNA–treated iBMMs/human proarteriogenic Mo/MΦs 24 hours after transfection (*n* = 5/group). Cells were blocked using FcR blocking reagent and stained with Nrp1-PeVio770 antibody (Miltenyi Biotec). Expression was determined using a MACSQuant flow cytometer with a relevant isotype control for setting gates.

Production of soluble Ang-1 and Vegfa was quantified in si*Htatip2-* and si*Control*-iBMMs by flow cytometry. Briefly, following siRNA transfection, culture media were replaced with normal iBMM media (IMDM, 20% FCS, 1% antibiotic/antimycotic, 50 ng/mL M-CSF) containing 0.1% GolgiPlug (BD Biosciences) for 24 hours. Cells were then harvested from culture and fixed in 2% paraformaldehyde for 15 minutes, followed by permeabilization using Perm Buffer I (BD Biosciences). Cells were stained with rabbit anti–mouse Vegf antibody (Abcam) and rat anti–mouse Angiopoietin-1 antibody (R&D Systems), followed by secondary staining with goat anti–rabbit AlexaFluor488 and donkey anti-rat DyLight405 antibodies (Supplemental Table 10). Staining was quantified using flow cytometry with appropriate isotype controls for gating.

### Collection of cell-conditioned media

In total, 2.5 × 10^5^ iBMMs/human Mo were treated with either *Htatip2* or *Control* siRNA as outlined previously. Following transfection, media were replaced with normal iBMM (IMDM, 20% HI serum, 1% antibiotic/antimycotic, 50 ng/mL M-CSF) or normal Mo media (RPMI1640, 10% HI serum, 1% L-glutamine, 1% antibiotic/antimycotic) for 24 hours. The culture supernatant was recovered from wells and centrifuged at 2,000*g* to remove cell debris (4°C for 10 minutes). 

### SMC XTT assay

Human SMCs (Lonza) were seeded onto a 96-well plate at a density of 1 × 10^3^/well and incubated for 24 hours in 100 μL of media conditioned previously by si*Htatip2* or si*Control*-iBMMs/human Mo. The cells were incubated with 1 mg/mL XTT for 4 hours, following which absorbance was measured at 450 nm using a plate reader (Filter Max F5, Molecular Devices), with a reference wavelength of 620 nm. Each assay was performed with 3 technical repeats.

### Blocking Ang-1 and Nrp1 activity in siHtatip2-iBMMs

In total, 2.5 × 10^5^ iBMMs were treated with either si*Htatip2* or si*Control* siRNA as outlined previously. After 18 hours, transfection media were replaced with normal iBMM culture media supplemented with 100 μg/mL anti-Tie2 (AF762, R&D Systems) or 30μg/mL anti-Nrp1 (AF566, R&D Systems); cells were incubated for 1 hour. For in vitro angiogenesis assays, cells were then tested as outlined previously. For in vitro arteriogenesis assays, cell culture media were harvested and analyzed using SMC XTT assays as described above.

### Western blot analysis of pErk expression

Total protein was isolated from Nrp1-blocked si*Htatip2*-iBMMs using RIPA buffer according to the manufacturer’s instructions (Thermo Fisher Scientific). Protein extract was quantified by Pierce BCA protein assay kit (Thermo Fisher Scientific) and measured using a SpectraMax340 plate reader. Protein concentrations were normalized, and cells were resuspended in SDS sample buffer prior to SDS-PAGE separation. Blots were blocked in 5% NFDM and stained for pErk or Histone H3 overnight. Secondary antibodies were HRP-conjugated horse anti–mouse IgG and goat anti–rabbit IgG (Supplemental Table 10). Protein bands were visualized using Novex ECL Chemiluminescent Substrate reagent kit (Thermo Fisher Scientific) on a Bio-Rad ChemiDoc MP Imaging system. Densiometric analysis of protein bands was performed using ImageJ software (NIH), and pErk protein levels were normalized to those of Histone H3 for each sample.

### HUVEC-fibroblast coculture angiogenesis assay

This assay is an adaptation of that previously reported (49). Briefly, human dermal fibroblasts (<P10, Lonza) were added to 24-well culture plates at a concentration of 2.4 × 10^4^ cells/well and cultured for 4 days to form a monolayer. HUVECs (<P3) were resuspended in EC growth media (Promocell) and added to the fibroblast containing wells at a concentration of 2.4 × 10^4^ cells/well. After 6 hours, media were aspirated from wells and replaced with growth media containing 2.4 × 10^4^ Tie2- or GFP-iBMMs per well or growth media supplemented with 100 ng/mL murine Vegf164. Plates were incubated under standard conditions (37°C, 21% O_2_, 5% CO_2_) for 14 days, with media replaced every 48 hours. For visualization of tubule formation, cells were first fixed in 70% ethanol. Following this, 0.3% hydrogen peroxide (v/v in 100% methanol) was applied to block endogenous alkaline phosphatase activity. Cells were incubated with mouse anti-human CD31 (R&D systems) and labeled endothelial tubes stained with alkaline-phosphatase–conjugated rabbit anti-mouse secondary antibody (Novus Biologicals) (Supplemental Table 10). Alkaline phosphatase activity was detected using BCIP/NBT substrate. Tubule formation was captured by Nikon COOLPIX990 camera and quantified by automated image processing (Image-Pro Plus). Specific information pertaining to all antibodies used in this manuscript are detailed in Supplemental Table 9.

### Mo chemoattractant protein-1 ELISA

Quantification of cell culture supernatant Mo chemoattractant protein 1 (MCP1) levels secreted by Tie2-iBMMs and GFP-iBMMs was assessed using a commercially available Quantikine ELISA kit (R&D Systems). The assay was carried out according to manufacturer’s instructions, and conditioned media samples were diluted 1:10 in calibrator diluent. Absorbance was measured at 450 nm using a SpectraMax340 spectrophotometer. MCP1 concentrations were interpolated from the MCP1 standard curve using a 4-parameter fit.

### Statistics

The statistical details of all experiments can be found in the appropriate figure legends. Technical and experimental repeats were conducted to ensure that experiments achieved at least 80% statistical power. All data were tested for normality and equal variance. Statistical analyses were carried out using Mann-Whitney *U* test for unpaired data or Wilcoxon rank test for paired data. Student’s 2-tailed *t* test or Wilcoxon rank test for paired data, Holm-Šidák test for paired multiple comparison tests, 1- way or 2-way (for repeated measures analysis of HLI) ANOVA for multiple groups with appropriate post hoc Bonferroni, or Tukey analyses were used. Data from replicate experiments are represented as mean ± SEM. A *P* value of less than 0.05 was considered statistically significant.

All tests were carried out using GraphPad Prism v8 software. Flow cytometric data were analyzed using FlowJo vX software. Unless stated otherwise, data are presented as mean ± SEM.

### Study approval

Patient whole-blood samples were obtained under local ethical committee approval with written informed consent from each individual. For experiments using mouse models, all experimental protocols and husbandry procedures were approved by the Home Office and carried out in accordance with ARRIVE guidelines.

### Data availability

Raw and normalized data files generated from the aforementioned microarray are deposited at http://www.ncbi.nlm.nih.gov/geo/query/acc.cgi?acc=GSE84626 Values for all data points in graphs are reported in the Supporting Data Values file.

## Author contributions

ASP, FEL, AM, and KN performed experiments; FEL and ASP analyzed results and constructed the figures; PS, OL, RS, MLS, and MDP provided intellectual input into the cellular and animal studies; ASP, FEL, AS, and BM designed the research and wrote the paper with input from all coauthors, who read, edited, and approved the final manuscript. Order of co–first authors was determined based on the amount of work contributed.

## Supplementary Material

Supplemental data

undefined

## Version 1 10/17/2023

In-Press Preview

## Version 2 12/22/2023

Electronic publication
