# Supplementary material for: HTATIP2 regulates arteriogenic activity in monocytes from patients with limb ischemia
Source: JCI Insight. 2023 Dec 22;8(24):e131419. doi: 10.1172/jci.insight.131419 (PMC10807724; doi:10.1172/jci.insight.131419)
Supplement: Supplemental data [file jciinsight-8-131419-s203.pdf]

Supplemental Figures

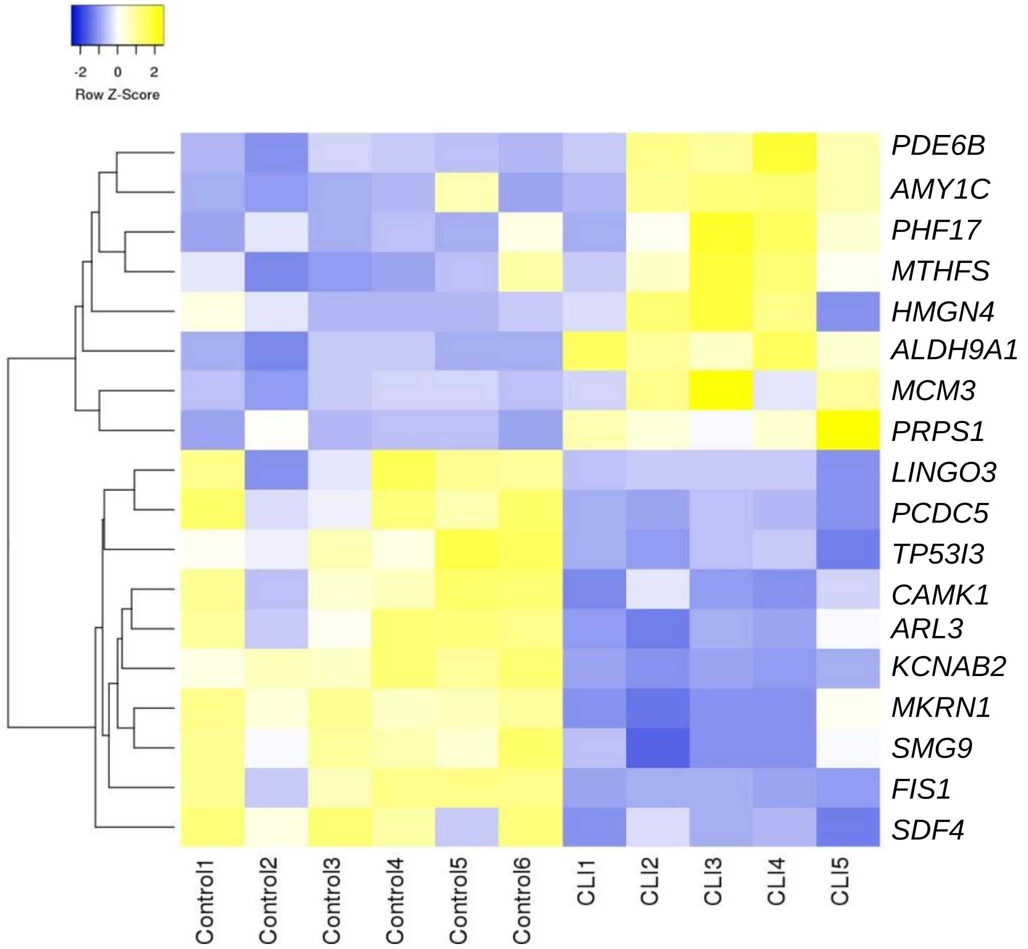

Figure S1 Top differentially-expressed genes between matched control and CLTI patient pro-arteriogenic Mo/MΦs. Heatmap depicting the top 20 differentially-expressed genes between matched control (n=6) and CLTI patient (n=5) pro-arteriogenic Mo/MΦs. Columns represent biological replicates, and rows represent genes. Gene expression is illustrated using pseudocolour scale (-2 to 2) with yellow representing high expression, and the blue representing low expression. Clustering between genes is determined using Euclidean distance metric. Mo/MΦ, monocyte/macrophage; CLTI, chronic limb threatening ischemia.

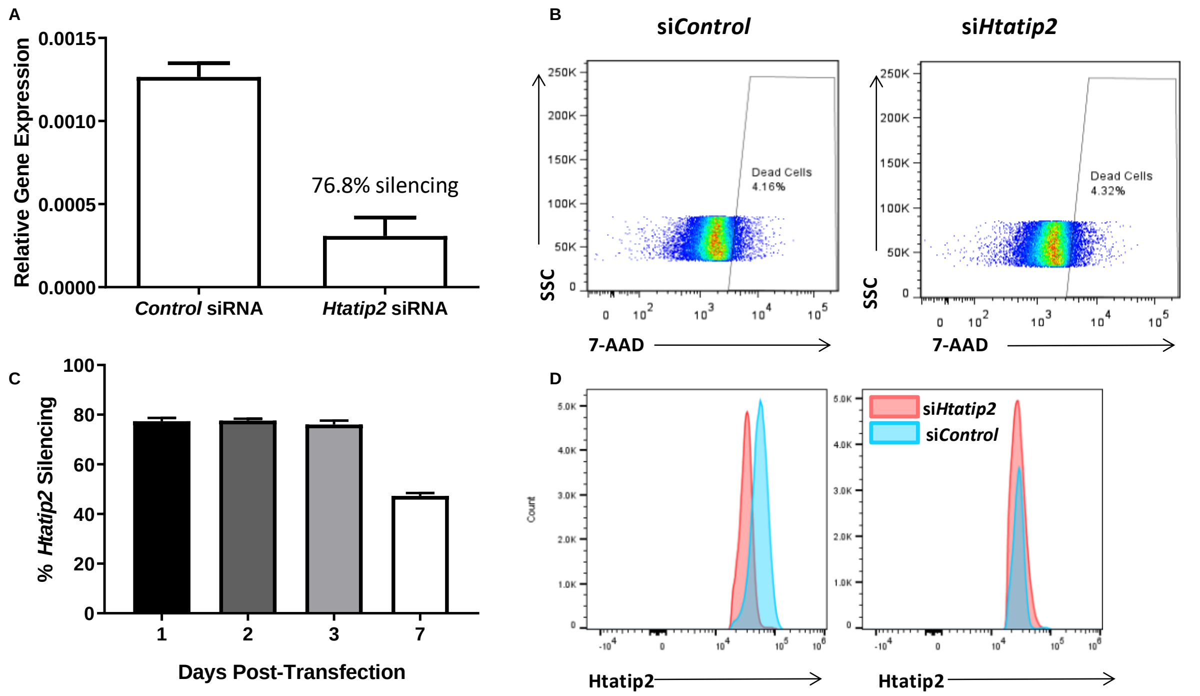

Figure S2 Optimization of *Htatip2* siRNA transfection. (A) The efficiency of siRNA silencing of *Htatip2* expression in iBMMs was determined by qPCR. (B) Cell death in siRNA-transfected iBMMs was quantified by 7-AAD staining using flow cytometry. (C) The duration of *Htatip2* silencing was quantified by qPCR, with relative *Htatip2* expression normalized to *Actb* and *Gapdh* expression over 7days following siRNA transfection. (D) Duration of Htatip2 silencing as determined by flow cytometry at day 3 (left) and day 7 (right) post-transfection in si*Htatip2*-treated Tie2-iBMMs (red peak) compared with si*Control*-treated cells (blue peak). Data information: In (A) and (C) Ct values of *Htatip2* mRNA was normalized to expression of *Actb* and *Gapdh* (n=3/group). The percentage of *Htatip2* mRNA silencing achieved was determined by comparing with cells transfected with a control siRNA. *Htatip2*, HIV-1 Tat interactive protein-2; iBMM, immortalized bone marrow macrophage; 7-AAD, 7-aminoactinomycin D; *Actb*, β-actin.

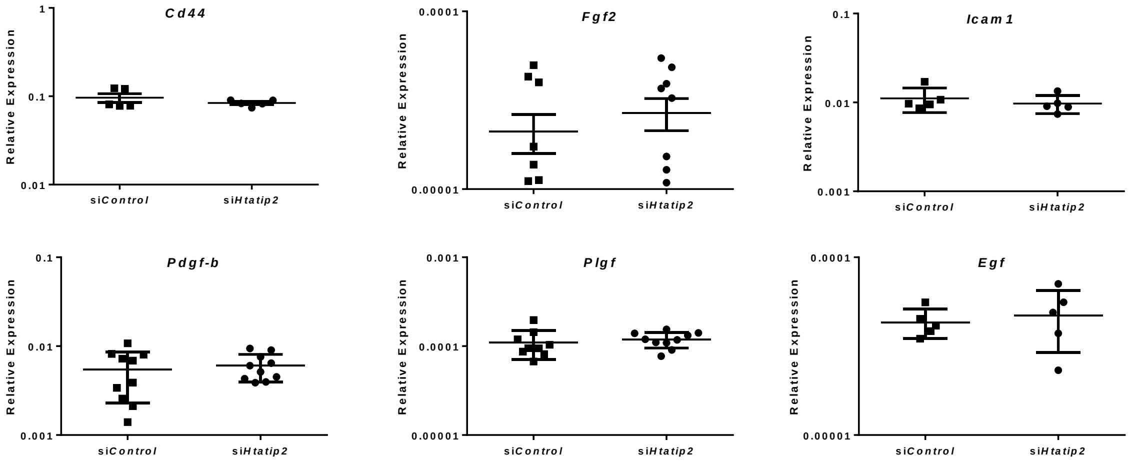

Figure S3 mRNA expression of pro-arteriogenic factors following *Htatip2* silencing in Tie2-iBMMs. Relative mRNA expression of *Cd44*, *Fgf2*, *Icam1*, *Pdgf-b*, *Plgf* and *Egf* in si*Control* and si*Htatip2* Tie2-iBMMs (n=5-10/group). Expression is normalized to expression of *Actb* and *Gapdh* reference genes. P>0.05 by Mann-Whitney test. *Htatip2*, HIV-1 Tat interactive protein-2; *Plgf*, Placental growth factor; iBMM, immortalized bone marrow macrophage.

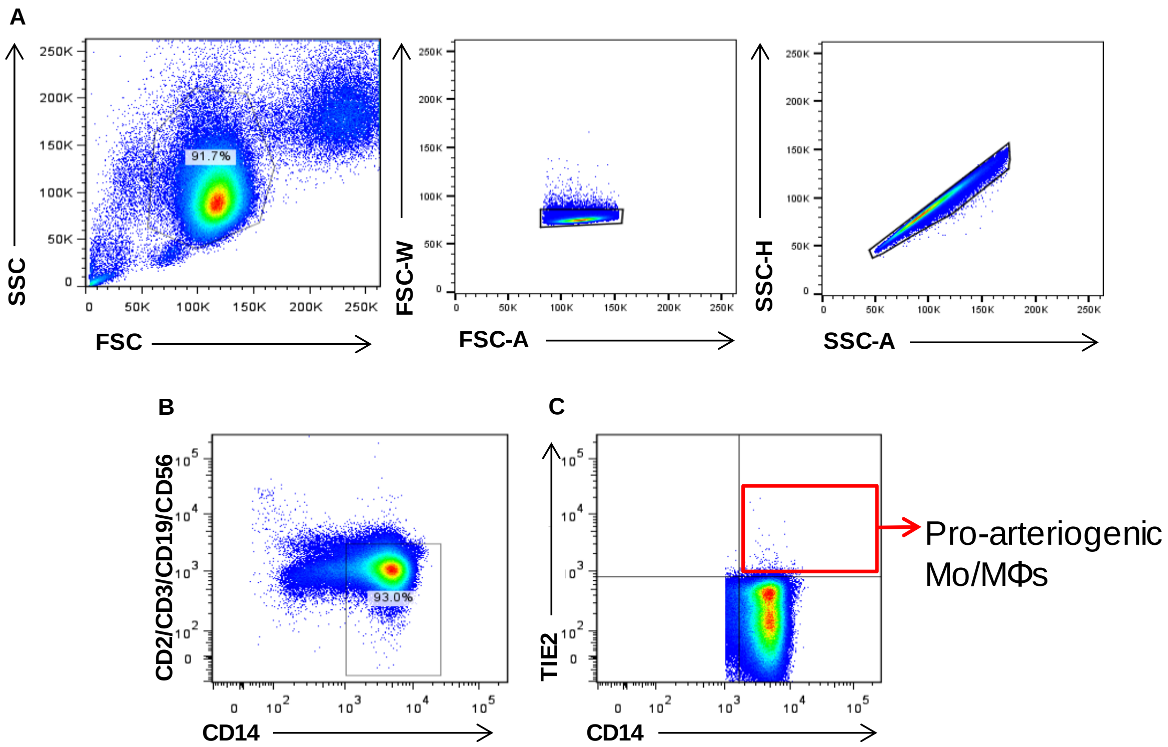

Figure S4 Gating strategy for pro-arteriogenic Mo/MΦs isolation by FACS. (A and B) Pro-arteriogenic Mo/MΦs were isolated by FACS following selection of single cell events and subsequent selection of CD14$^+$ and CD2/CD3/CD19/CD56$^-$ cells based on gating set by appropriate isotype controls. (C) TIE2$^+$ monocytes were stringently selected for based on isotype control fluorescence for gating. Mo/MΦ, monocyte/macrophage; FSC, forward scatter; SSC, side scatter.

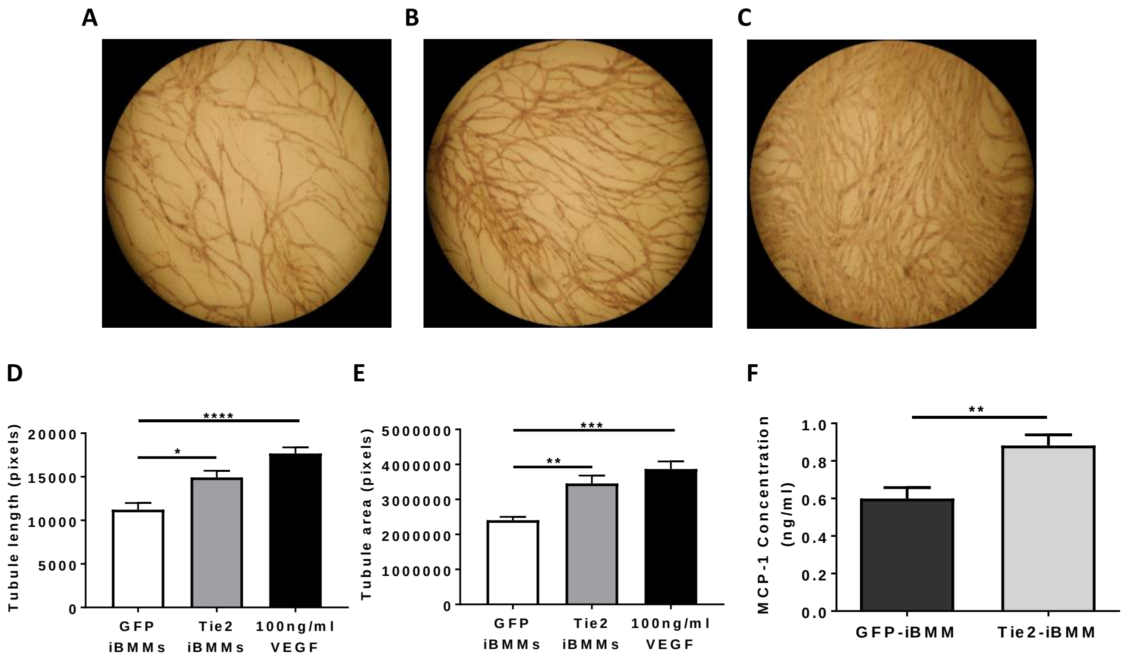

Figure S5 Tie2-iBMMs have pro-angiogenic function. (A-C) Light microscopy images of CD31$^+$ HUVEC tubule formation following co-culture with GFP-iBMMs (A), Tie2-iBMMs (B) and 100ng/ml VEGF (C) using a 4X objective lens. (D and E) Length (D) and area (E) of endothelial cell tubule formation following co-culture with GFP-iBMMs, Tie2-iBMMs or 100ng/ml VEGF (n=12/group). (F) Quantification of GFP-iBMM and Tie2-iBMM-secreted MCP-1 by ELISA (n=6/group). Data in D&E are presented as mean ± SEM. Data in F represents mean ± SD. *P<0.05 **P<0.01 ***P<0.001 ****P<0.0001 by t-test or Kruskal-Wallis and Dunn's Multiple Comparison Test. iBMM, immortalized bone marrow macrophage; MCP-1, monocyte chemoattractant protein-1.

Supplemental Tables

Table S1: Table of top 20 differentially expressed genes between CLTI and control patient pro-arteriogenic Mo/MΦs

Gene	Fold-Change	P-value
FIS1	-139.17	0.0278
ARL3	-82.77	0.0051
KCNAB2	-82.74	0.0029
TP53I3	-81.17	0.0048
SMG9	-74.99	0.0057
MKRN1	-60.23	0.0119
PDCD5	-57.97	0.0059
SDF4	-51.05	0.0092
LINGO3	-49.83	0.0113
CAMK1	-47.72	0.0162
MTHFS	42.29	0.0026
PAPD7	43.62	0.0151
TMEM106A	46.81	0.0016
ALDH9A1	49.91	0.0032
PHF17	53.12	0.0019
PRPS1	60.37	0.0031
MCM3	69.17	0.0016
HMGN4	73.99	0.0015
AMY1C	75.15	0.0041
PDE6B	166.47	<0.0001

Table S2: Top differentially expressed angiogenesis-associated genes between CLTI and control patient pro-arteriogenic Mo/MΦs

Gene	Fold-Change	P-value
HIPK1	-34.72	0.0003
PML	-13.36	0.0192
PTEN	-7.80	0.0343
TNFRSF12A	-6.13	0.0383
LECT1	-6.04	0.0084
EDNRA	-4.78	0.0175
GTF2I	4.17	0.0495
MAPK3	5.48	0.0288
DDAH1	7.32	0.0322
HTATIP2	9.24	0.0466
ROCK1	11.50	0.0231
NF1	15.41	0.0386

Table S3: Most significantly enriched functional groups in differentially expressed genes between CLTI patients and controls in TIE2^{-ve} Mo/MΦs

Classification	Group	*P*-value	No. of genes
GO biological process	GO:0043982: histone H4-K8 acetylation	0.0001	5
GO molecular function	GO:0010485: H4 histone acetyltransferase activity	<0.0001	5
GO cellular component	GO:0043229: intracellular organelle	0.0005	346
KEGG pathways	Protein processing in endoplasmic reticulum	0.0383	15
IPA pathways	Retinoic acid Mediated Apoptosis Signaling	0.0084	7
Network	Embryonic Development, Organismal Development, Tissue Development		27

Table S4: Most significantly enriched functional groups in differentially expressed genes between CLTI patients and controls in pro-arteriogenic (TIE2^{+ve}) Mo/MΦs

Classification	Group	*P*-value	No. of genes
GO biological process	GO:0071704: organic substance metabolic process	<0.0001	233
GO molecular function	GO:0050897: cobalt ion binding	0.0001	3
GO cellular component	GO:0043226: organelle	<0.0001	372
KEGG pathways	Ubiquitin mediated proteolysis	0.0129	10
IPA pathways	Thyroid Cancer Signaling	0.0107	5
Network	Cell Morphology, Cellular Assembly and Organization, Cell Cycle		31

Table S5: Patient data for microarray samples

Characteristic		CLI patients (n=5)	Control patients (n=6)
Age (range)		53-92 years (mean 73.8 years)	62-86 years (mean 72.6 years)
Rutherford score	*IV*	2 (40%)	0 (100%)
	V	3 (60%)	0 (100%)
Male		2 (40%)	6 (100%)
Positive smoking history		4 (80%)	2 (33%)
Hypertension		3 (60%)	3 (50%)
Hyperlipidemia		5 (100%)	3 (50%)

Table S6: qPCR primer details

Gene target	Accession number	Forward primer sequence	Reverse primer sequence
ACTB	NM_0011	CCAACCGCGAGAAGATGA	CCAGAGGCGTACAGGGATAG
RPLPO	NM_001002	TCTACAACCCTGAAGTGCTTGAT	CAATCTGCAGACAGACACTGG
MAPK3	NM_002746	CCCTAGCCCAGACAGACATC	GCACAGTGTCCATTTTCTAACAGT
HTATIP2	NM_001098523	TCGTCTTTCAAAGTAATCCTTGAGT	GCGATAATCACAATAGGCAAAA

Table S7: siRNA oligonucleotide sequences used for silencing *Htatip2*

siRNA	Target Sequence
Control	AATTCTCCGAACGTGTCACGT
Htatip2-1	TTCGAGGAGGAAGCTTATAAA
Htatip2-2	CAGGTTTATATCATAGACTTA
Htatip2-3	CAGGAGGGTGCAAACATTTCA
Htatip2-4	AGCCAAGGTTGAAGAATTAAA

Table S8: siRNA oligonucleotide sequences used for silencing *HTATIP2*

siRNA	Target Sequence
CONTROL	AATTCTCCGAACGTGTCACGT
HTATIP2-1	CACTTACCGACCAATCTTT
HTATIP2-2	CTAGCAATGAGACATAAAT
HTATIP2-3	CCTCCCTAAACAAGCACAA

Table S9: Antibody details

Antigen	Conjugate	Supplier	Assay Concentration/Dilution
TIE2	PE	R&D Systems #FAB3131P	1:10
CD2	V450	BD Biosciences #644485	0.5µg/ml
CD3	V450	BD Biosciences #560351	0.5µg/ml
CD14	FITC	BD Biosciences #555397	2µg/ml
CD19	V450	BD Biosciences #560353	0.5µg/ml
CD56	V450	BD Biosciences #560360	0.5µg/ml
NRP1	APC	Miltenyi Biotec #130-113-515	7.5µg/ml
Tie2	PE	Biologend #124008	5µg/ml
Nrp1	APC	Miltenyi Biotec #130-105-659	7.5µg/ml
Vegfa	Unconjugated	Abcam #ab52917	3µg/ml
Angiopoietin-1	APC	AssayPro #32215-05161	1.5µg/ml
HTATIP2	Unconjugated	Abcam #ab177961	1.75µg/µl
Laminin	AlexaFluor488	Novus Biologicals #NB300-144G	20µg/ml
CD31-PECAM	Unconjugated	BD Pharminogen #553370	5µg/ml
α-SMA	Cy3	Sigma Aldrich #C6198	10µg/ml
pErk	Unconjugated	Cell Signalling Technology #9101S	1:1000
Histone H3	Unconjugated	Cell Signalling Technology #9717	1:1000
CD31-PECAM	Unconjugated	R&D Systems #BBA7	0.5µg/ml
Rabbit anti-mouse IgG1	Alkaline phosphatase	Novus Biologicals #NBP1-72672	2µg/ml
Horse anti-mouse IgG	HRP	Cell Signalling Technology #7076	1:2000
Goat anti-rabbit IgG	HRP	Cell Signalling Technology #7074	1:2000
Donkey anti-rat IgG	Cy3	JacksonImmunoResearch #712-166-150	75µg/µl
Donkey anti-rabbit IgG	APC	JacksonImmunoResearch #711-136-152	75µg/µl
